# Supplementary material for: Suppression of Choroidal Neovascularization and Fibrosis by a Novel RNAi Therapeutic Agent against (Pro)renin Receptor
Source: Mol Ther Nucleic Acids. 2019 May 28;17:113–25. doi: 10.1016/j.omtn.2019.05.012 (PMC6599885; doi:10.1016/j.omtn.2019.05.012)
Supplement: Document S1. Figures S1–S4 and Table S1 [file mmc1.pdf]

OMTN, Volume *17*

Supplemental Information

Suppression of Choroidal Neovascularization and Fibrosis by a Novel RNAi Therapeutic Agent against (Pro)renin Receptor

Ye Liu, Atsuhiro Kanda, Di Wu, Erdal Tan Ishizuka, Satoru Kase, Kousuke Noda, Atsuhiro Ichihara, and Susumu Ishida

Supplemental information

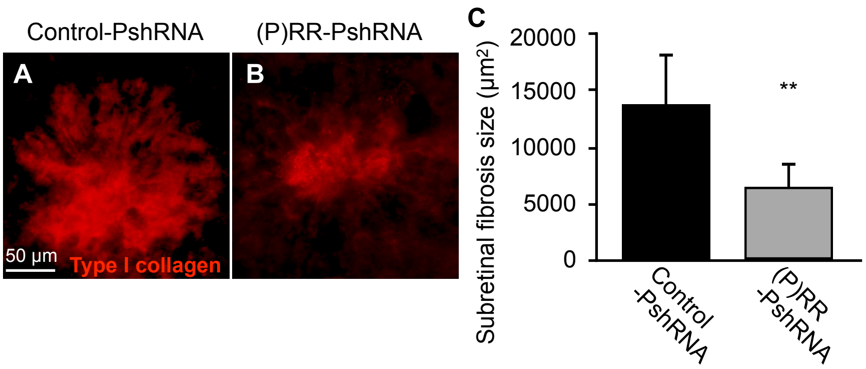

Figure S1. Suppression of subretinal fibrosis at 21 days after laser photocoagulation by (P)RR-PshRNA

(A, B) Representative micrographs of subretinal fibrosis lesions (type I collagen, *red*) in the RPE-choroid flat mounts at post-laser day 21 from mice treated with 100 pmol control-PshRNA (A) or (P)RR-PshRNA (B). Scale bar, 50 μm. (C) Quantification analysis of the size of subretinal fibrosis [control-PshRNA = 13,695 ± 4,566 μm^2, (P)RR-PshRNA = 6,381 ± 2,236 μm^2]. $**p < 0.01$ (n = 5).

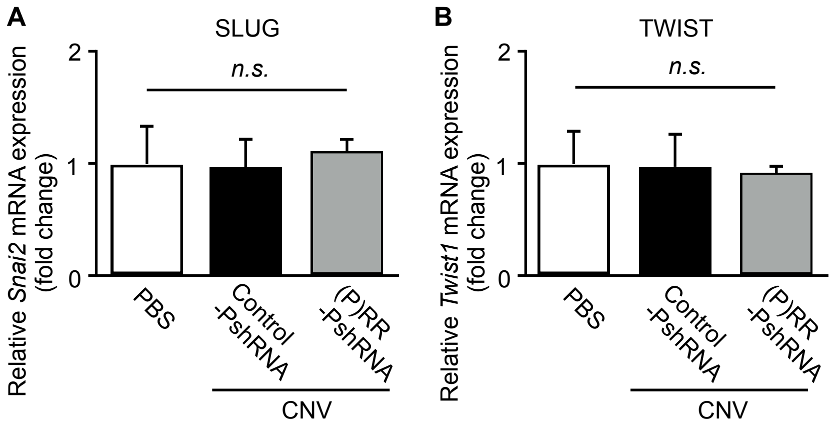

Figure S2. Neither SNAI2 nor TWIST1 is associated with CNV induction in mice

(A, B) Relative mRNA expression levels of *Snai2* (A) and *Twist1* (B) in the RPE-choroid complex from untreated normal mice (control) and CNV mice treated with 100 pmol control-PshRNA or (P)RR-PshRNA. n.s., not significant (n = 6).

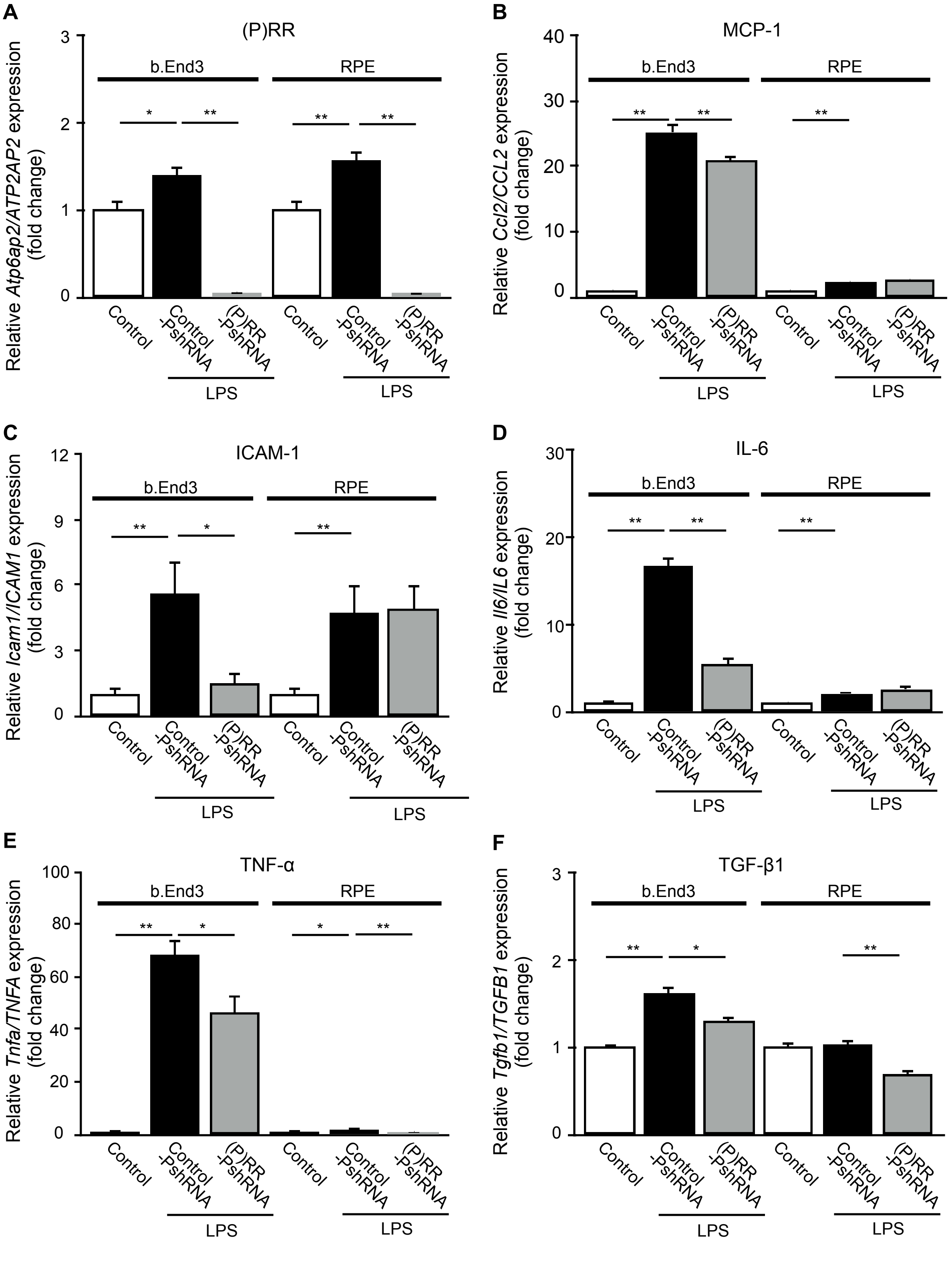

Figure S3. Blockade of inflammatory responses by (P)RR-PshRNA in LPS-stimulated endothelial cells and RPE cells

(A-E) Gene expression levels of inflammatory molecules *Atp6ap2/ATP6AP2* (A), *Ccl2/CCL2* (B), *Icam1/ICAM1* (C), *Il6/IL6* (D), *Tnfa/TNFA* (E) and profibrotic cytokine *Tgfb1/TGFB1* (F) in 1 nM control-PshRNA or (P)RR-PshRNA transfected mouse microvascular endothelial cells stimulated with 10 ng/ml LPS for 8 hours and 1 nM control-PshRNA or (P)RR-PshRNA transfected human RPE cells stimulated with 10 ng/ml LPS for 12 hours. *$p < 0.05$, **$p < 0.01$ (n = 6).

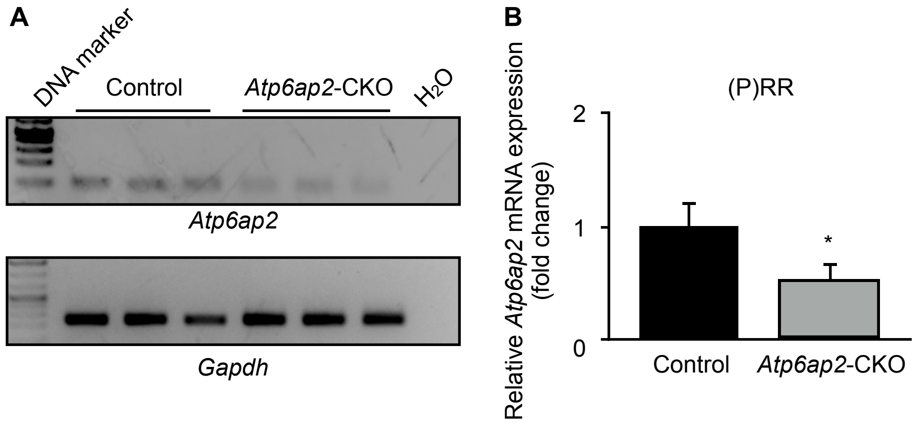

Figure S4. Gene expression of *(P)RR/Atp6ap2* in the RPE-choroid complex of *(P)RR*/*Atp6ap2*-CKO mice

(A) Gene expression analysis of *(P)RR/Atp6ap2* in the RPE-choroid complex of control and *(P)RR*/*Atp6ap2*-CKO mice. *Gapdh* was used as an internal control. (B) Quantification analysis of the relative expression of *(P)RR*/*Atp6ap2*. *$p < 0.05$ (n = 3).

Table S1. Primer sequences used in quantitative RT-PCR

Target gene	Sequence
Mouse	
(P)RR/Atp6ap2	forward 5'- CCTCATTAGGAAGACAAGGACTATCC -3'
	reverse 5'- GGGTTCTTCGCTTGTTTTGC -3'
Ccl2	forward 5'- TTGGCTCAGCCAGATGCA -3'
	reverse 5'- CCTACTCATTGGGATCATCTTGC -3'
Icam1	forward 5'- CCTGTTTCCTGGCTCTGAAG -3'
	reverse 5'- GTCTGCTGAGACCCCTCTTG -3'
Il6	forward 5'- CACAGAGGATACCACTCCCAACA -3'
	reverse 5'- TCCACGATTTCCCAGAGAAACA -3'
Tnfa	forward 5'- GGTGCCTATGTCTCAGCCTCTT -3'
	reverse 5'- CGATCACCCCGAAGTTCAGTA -3'
Emr1	forward 5'- CTTTGGCTATGGGCTTCCAGTC -3'
	reverse 5'- GCAAGGAGGACAGAGTTTATCGTG -3'
Tgfb1	forward 5'- CAGTGGCTGAACCAAGGAGAC -3'
	reverse 5'- ATCCCGTTGATTTCCACGTG -3'
Acta2	forward 5'- TCTGTAAGGCCGGCTTTGC -3'
	reverse 5'- TGTCCCATTCCCACCATCA -3'
Col1a1	forward 5'- TGACTGGAAGAGCGGAGAGT -3'
	reverse 5'- GACGGCTGAGTAGGGAACAC -3'
Fn1	forward 5'- GTCAGTGTCTCCAGTGTCTAC -3'
	reverse 5'- TGGCTTGCTGGCCAATCAGT -3'
Snai1	forward 5'- CACACGCTGCCTTGTGTCT -3'
	reverse 5'- GGTCAGCAAAAGCACGGTT -3'
Snai2	forward 5'- CAGCGAACTGGACACACACA -3'
	reverse 5'- ATAGGGCTGTATGCTCCCGAG -3'
Twist1	forward 5'- GGACAAGCTGAGCAAGATTCA -3'
	reverse 5'- CGGAGAAGGCGTAGCTGAG -3'
Gapdh	forward 5'- AGGTCGGTGTGAACGGATTTG -3'
	reverse 5'- TGTAGACCATGTAGTTGAGGTCA -3'
Human	
(P)RR/ATP6AP2	forward 5'- AGGCAGTGTCATTTCGTACC -3'
	reverse 5'- GCCTTCCCTACCATATACACTC -3'
CCL2	forward 5'- CGCCTCCAGCATGAAAGTCT -3'
	reverse 5'- ATGAAGGTGGCTGCTATG -3'
ICAM1	forward 5'- GCAAGCTCCCAGTGAAATGCAAAC -3'
	reverse 5'- TGTCTACTGACCCCAACCCTTGATG -3'
IL6	forward 5'- CCACTCACCTCTTCAGAACG -3'
	reverse 5'- CATCTTTGGAAGGTTCAGGTTG -3'
TNFA	forward 5'- ACTTTGGAGTGATCGGCC -3'
	reverse 5'- GCTTGAGGGTTTGCTACAAC -3'
TGFB1	forward 5'- GCCCTGGACACCAACTATTG -3'
	reverse 5'- CGTGTCCAGGCTCCAAATG -3'
GAPDH	forward 5'- CCTGGCCAAGGTCATCCATG -3'
	reverse 5'- GGAAGGCCATGCCAGTGAGC -3'
