# Supplementary material for: Suppression of Choroidal Neovascularization and Fibrosis by a Novel RNAi Therapeutic Agent against (Pro)renin Receptor
Source: Mol Ther Nucleic Acids. 2019 May 28;17:113–25. doi: 10.1016/j.omtn.2019.05.012 (PMC6599885; doi:10.1016/j.omtn.2019.05.012)
Supplement: Document S2. Article plus Supplemental Information [file mmc2.pdf]

Molecular Therapy
Nucleic Acids
Original Article

AMERICAN SOCIETY of GENE & CELL THERAPY

# Suppression of Choroidal Neovascularization and Fibrosis by a Novel RNAi Therapeutic Agent against (Pro)renin Receptor

Ye Liu,[1] Atsuhiro Kanda,[1] Di Wu,[1] Erdal Tan Ishizuka,[1] Satoru Kase,[1] Kousuke Noda,[1] Atsuhiro Ichihara,[2] and Susumu Ishida[1]

[1]Laboratory of Ocular Cell Biology and Visual Science, Department of Ophthalmology, Faculty of Medicine and Graduate School of Medicine, Hokkaido University, Sapporo, Hokkaido 060-8638, Japan; [2]Department of Endocrinology and Hypertension, Tokyo Women's Medical University, Tokyo 162-8666, Japan

**The receptor-associated prorenin system refers to the pathogenic mechanism whereby prorenin binding to (pro)renin receptor [(P)RR] dually activates the tissue renin-angiotensin system (RAS) and RAS-independent signaling, and its activation contributes to the molecular pathogenesis of various ocular diseases. We recently developed a new single-stranded RNAi agent targeting both human and mouse *(P)RR* ((P)RR-proline-modified short hairpin RNA [(P)RR-PshRNA]), and confirmed its therapeutic effect on murine models of ocular inflammation. Here, we investigated the efficacy of (P)RR-PshRNA against laser-induced choroidal neovascularization (CNV) and subretinal fibrosis, both of which are involved in the pathogenesis of age-related macular degeneration (AMD). Administration of (P)RR-PshRNA in mice significantly reduced CNV formation, together with the expression of inflammatory molecules, macrophage infiltration, and extracellular signal-regulated kinase (ERK) 1/2 activation. In addition, (P)RR-PshRNA attenuated subretinal fibrosis, together with epithelial-mesenchymal transition (EMT)-related markers including phosphorylated SMAD2. The suppressive effect of (P)RR-PshRNA is comparable with aflibercept, an anti-vascular endothelial growth factor drug widely used for AMD therapy. AMD patient specimens demonstrated (P)RR co-localization with phosphorylated ERK1/2 in neovascular endothelial cells and retinal pigment epithelial cells. These results indicate that (P)RR contributes to the ocular pathogenesis of both inflammation-related angiogenesis and EMT-driven fibrosis, and that (P)RR-PshRNA is a promising therapeutic agent for AMD.**

## INTRODUCTION

Age-related macular degeneration (AMD) is the leading cause of irreversible blindness worldwide.[1] The wet (neovascular or exudative) type of AMD usually causes severe vision loss and is characterized by choroidal neovascularization (CNV), which extends from the choroid into the subretinal space and disrupts Bruch's membrane and the retinal pigment epithelium (RPE). The development of CNV is subsequently followed by subretinal fibrosis, which accounts for most cases of the severe vision loss in the late stage of AMD, leading to permanent photoreceptor damage and vision loss.[2,3] Among the several molecular players involved in the pathogenesis of CNV, vascular endothelial growth factor (VEGF)-A plays distinct roles in pathological angiogenesis and inflammation in AMD.[4] Anti-VEGF therapy has become the standard treatment for improving visual acuity; however, a large percentage of patients suffer from poor visual prognosis because of subretinal fibrosis.[5,6] Therefore, alternative therapeutic approaches, which can attenuate both CNV and subretinal fibrosis formation, would fulfill an unmet medical need in the treatment of AMD.

The renin-angiotensin system (RAS), a known key regulator of systemic blood pressure and water balance (circulatory RAS), has distinct roles in pathological angiogenesis and inflammation in various organs (tissue RAS).[7,8] (Pro)renin receptor [(P)RR, encoded by the *ATP6AP2* gene], which locates at the upstream of tissue RAS, binds with prorenin, leading to not only the activation of tissue RAS but also its intracellular signaling pathways through phosphorylation of extracellular signal-regulated kinase (ERK) 1/2, and regulates the expression of various pathogenic molecules including monocyte chemotactic protein (MCP)-1, also known as C-C motif chemokine ligand (CCL) 2, and intercellular adhesion molecule (ICAM)-1.[9,10] This dual activation of tissue RAS and the RAS-independent signaling, termed as the receptor-associated prorenin system (RAPS), was revealed to be involved in the molecular pathogenesis such as inflammation and pathological angiogenesis in various disorders.[9–13] In addition, activation of (P)RR increases the expression of transforming growth factor (TGF)-β1 and extracellular matrix proteins (e.g., type I collagen and fibronectin), leading to fibrotic changes in several renal cell lines and in the heart and kidney of hypertensive rats.[14–17] Importantly, we revealed the expression of RAPS

Received 31 January 2019; accepted 20 May 2019;
https://doi.org/10.1016/j.omtn.2019.05.012.

**Correspondence:** Atsuhiro Kanda, PhD, Laboratory of Ocular Cell Biology and Visual Science, Department of Ophthalmology, Faculty of Medicine and Graduate School of Medicine, Hokkaido University, N-15, W-7, Kita-ku, Sapporo, Hokkaido 060-8638, Japan
**E-mail:** kanda@med.hokudai.ac.jp


This is an open access article under the CC BY license (http://creativecommons.org/licenses/by/4.0/).

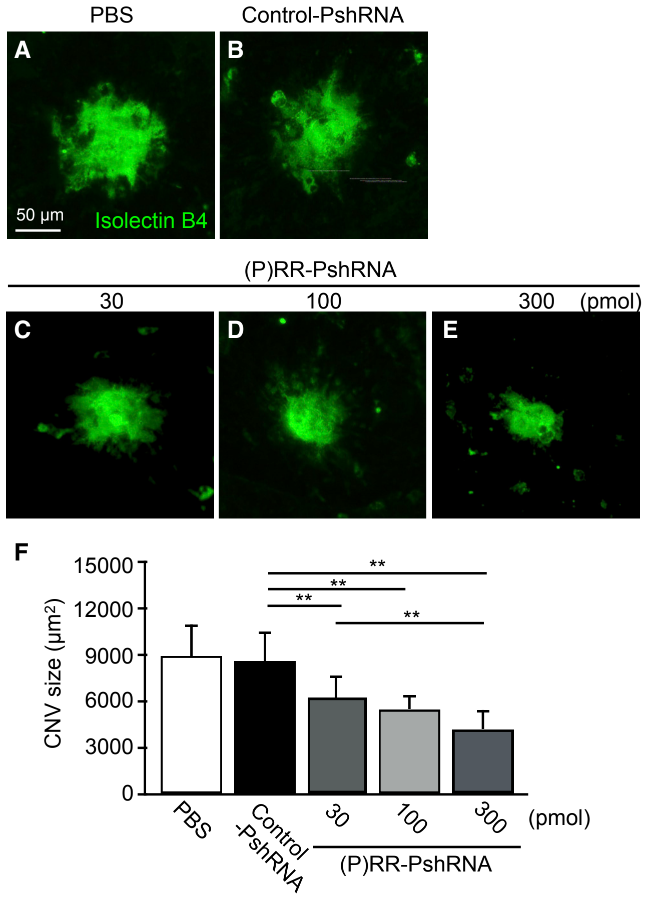


**Figure 1. Suppression of Laser-Induced CNV Formation by (P)RR-PshRNA**

(A–E) Representative micrographs of CNV lesions (isolectin B4, green) in the RPE-choroid flat mounts at post-laser day 7 from mice treated with (A) PBS, (B) 100 pmol control-PshRNA, or (C) 30, (D) 100, and (E) 300 pmol (P)RR-PshRNA, respectively. Scale bar, 50 μm. (F) Quantification analysis of the size of CNV [PBS = 8,966 ± 2,040 $\mu m^2$, 100 pmol control-PshRNA = 8,660 ± 1,819 $\mu m^2$, 30 pmol (P)RR-PshRNA = 6,234 ± 1,424 $\mu m^2$, 100 pmol (P)RR-PshRNA = 5,526 ± 898 $\mu m^2$, 300 pmol (P)RR-PshRNA = 4,245 ± 1,185 $\mu m^2$]. $^{**}p < 0.01$ (n = 5).

components, including (P)RR, in the surgically excised fibrotic tissues from patients with idiopathic epiretinal membrane and proliferative diabetic retinopathy,[18,19] indicating that (P)RR contributes to ocular fibrotic disorders.

RAS blockers including a direct renin inhibitor aliskiren have no effect on suppressing the (P)RR downstream signaling because of prorenin-(P)RR interaction.[20] Although (P)RR blocker (PRRB), a peptide with the structure of the handle region of the prorenin prosegment working as a decoy for (P)RR, is the only available agent to block prorenin-(P)RR binding,[21] it has several limitations (e.g., induction of immune response and protease resistance), which preclude its future clinical application. RNAi is a useful method of suppressing gene expression because of its high selectivity and potency; however, canonical double-stranded small interfering RNAs (siRNAs) have several problems including the activation of innate immunity via Toll-like receptors.[22] Recently, we developed a novel single-stranded RNAi agent ((P)RR-proline-modified short hairpin RNA [(P)RR-PshRNA]), which overcame these obstacles, and found that application of (P)RR-PshRNA to mice caused significant amelioration of acute (uveitic) and chronic (diabetic) models of ocular inflammation, by downregulating the expression of inflammatory molecules without adverse events *in vivo* and *in vitro*.[23] In this study, we investigated the ability of (P)RR-PshRNA to suppress inflammation-related angiogenesis and fibrosis together with underlying molecular mechanisms using a CNV mouse model, RPE and endothelial cell culture, and human AMD specimens.

## RESULTS

### Suppression of Laser-Induced CNV Formation by (P)RR-PshRNA

Previously, we reported that inhibition of angiotensin II type 1 receptor (AT1R) and (P)RR attenuated laser-induced CNV and inflammation using the animal model, and demonstrated that RAPS was involved in CNV formation through activation of its intracellular signaling pathways.[8,9] To examine whether intravitreal injection of (P)RR-PshRNA suppresses the formation of CNV, we quantified the CNV size stained by isolectin B4 in the flat mounts of the RPE-choroid complex, in which the CNV tissue arises. Notably, (P)RR-PshRNA injected into the vitreous cavity of murine eyes was revealed to deeply penetrate to the RPE and choroid.[23] Compared with PBS or 100 pmol control-PshRNA-treated eyes, 100 pmol (P)RR-PshRNA administration led to a significant decrease in the average CNV size at post-laser day 7 (Figures 1A, 1B, 1D, and 1F). These suppressive effects were observed in a dose-dependent manner (Figures 1C–1F).

### Inhibition of CNV-Related Inflammatory Molecule Expression, Macrophage Infiltration, and ERK1/2 Activation by (P)RR-PshRNA

To investigate the mechanisms by which (P)RR-PshRNA attenuates CNV formation, we examined expression levels of *(P)RR/Atp6ap2* and several major inflammatory molecules including *Ccl2*, *Icam1*, *Il6* (interleukin-6), and *Tnfa* (tumor necrosis factor-α), all of which are induced via (P)RR signaling[9–12,23] and also responsible for the pathogenesis of CNV.[24–27] Compared with normal mice, mRNA levels of *(P)RR/Atp6ap2*, *Ccl2*, *Icam1*, *Il6*, and *Tnfa* in the RPE-choroid complex of mice administrated with control-PshRNA significantly increased 3 days after laser photocoagulation (Figures 2A–2E). Intravitreal injection of (P)RR-PshRNA significantly inhibited mRNA expression of these inflammatory molecules as well as *(P)RR/Atp6ap2* (Figures 2A–2E). Macrophage infiltration to the choroid has been proposed to contribute to the pathogenesis of CNV through increased cytokine and chemokine expression.[28,29] To investigate whether administration of (P)RR-PshRNA affects the infiltration of macrophages, we assessed the expression of *Emr1* (F4/80), a mouse macrophage marker,[30] in the RPE-choroid complex. Intravitreal administration of (P)RR-PshRNA significantly reduced *Emr1* mRNA levels compared with the control-PshRNA (Figure 2F). Consistent with gene expression data, immunofluorescent staining using an

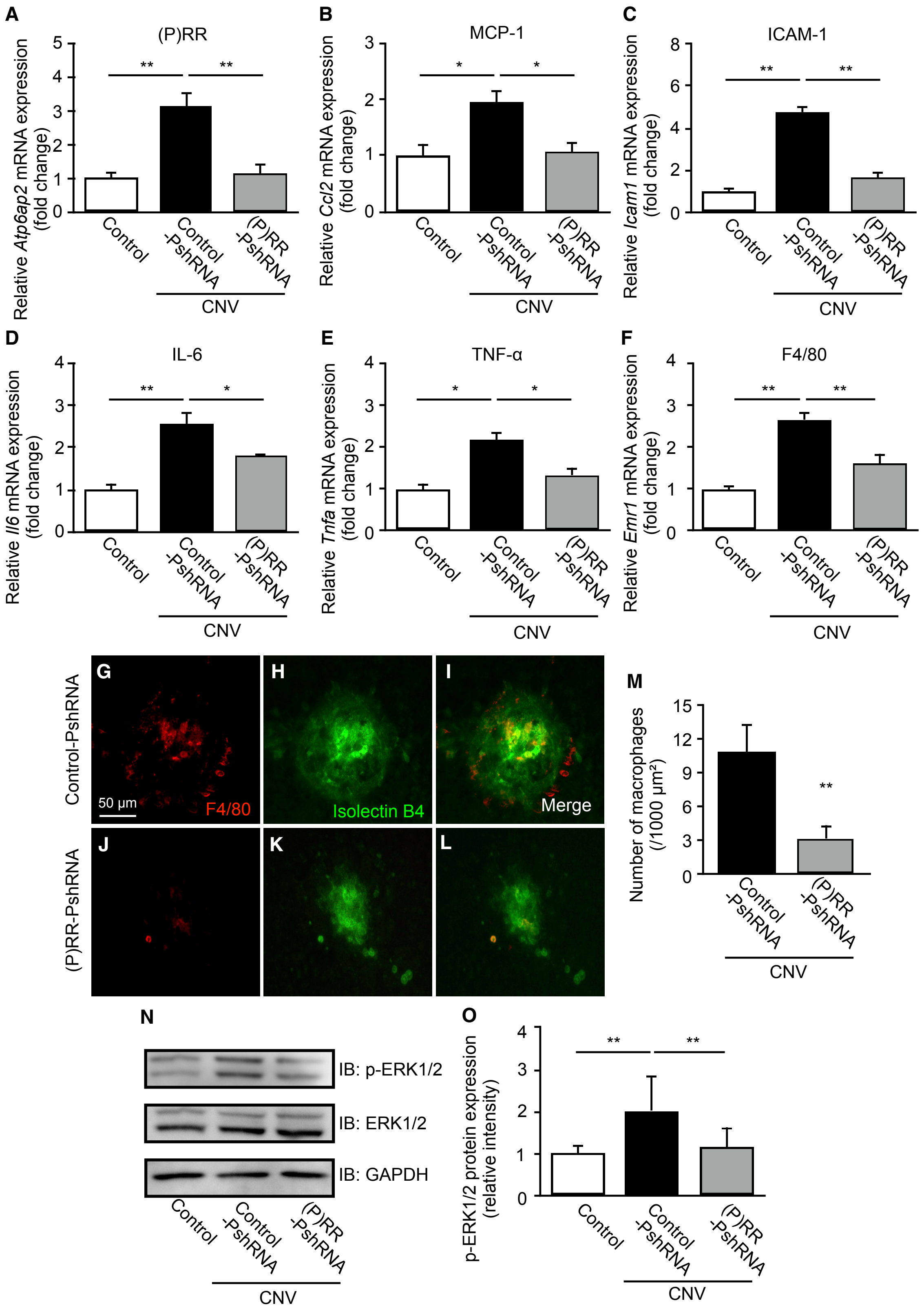


(legend on next page)

anti-F4/80 antibody and isolectin B4 for the CNV area showed that (P)RR-PshRNA administration significantly suppressed the number of F4/80-positive cells in the CNV area at 3 days after laser photocoagulation (Figures 2G–2M), suggesting that administration of (P)RR-PshRNA suppressed CNV-associated macrophage infiltration.

(P)RR has been reported to induce activation of ERK1/2 and production of various cytokines and growth factors *in vivo* and *in vitro*.[9,10,15,16,18] Therefore, we examined the inhibitory effects of (P)RR-PshRNA on phosphorylation of ERK1/2 in the RPE-choroid complex of mice with CNV. Consistent with our previous report in the CNV model,[9] CNV-related elevation of phosphorylated ERK1/2 levels in the RPE-choroid complex was reduced by inhibition of (P)RR with (P)RR-PshRNA at post-laser day 3 (Figures 2N and 2O), suggesting that administration of (P)RR-PshRNA can suppress upregulation of *(P)RR/Atp6ap2*, together with both inflammatory molecule expression and CNV formation via ERK1/2 activation.

### Suppression of Subretinal Fibrosis and Fibrotic Molecules via the ERK1/2-Mediated TGF-β/SMAD2/SNAIL Pathway by (P)RR-PshRNA

Subretinal fibrosis, a wound healing response that follows CNV in AMD, is accompanied by destruction of photoreceptors, RPE, and choroidal vessels, leading to severe vision loss.[5,6] The epithelial-mesenchymal transition (EMT) plays physiological roles in embryonic development and wound healing, whereas EMT dysfunction causes pathological reactions including carcinogenesis and fibrogenesis.[31–33] The activation of (P)RR has been shown to induce the expression of profibrotic molecules and EMT changes, contributing to cardiac fibrosis in genetic hypertension rats,[14] as well as mesangial fibrosis in renal cells.[15–17] To evaluate whether intravitreal injection of (P)RR-PshRNA suppresses CNV-associated subretinal fibrosis, composed mainly of type I collagen, we stained flat mounts of the RPE-choroid complex with an anti-type I collagen antibody to quantify the size of subretinal fibrosis at 7 and 21 days after laser. The average fibrosis size significantly decreased in CNV mice treated with (P)RR-PshRNA compared with controls at both evaluation points (Figures 3A–3C and S1A–S1C).

To further investigate the molecular mechanism for the attenuation of fibrosis, we analyzed mRNA expression levels of *Tgfb1* (TGF-β1), *Acta2* (α-smooth muscle actin [α-SMA]), *Col1a1* (type I collagen), and *Fn1* (fibronectin), all of which are representative EMT-related mesenchymal markers.[31–33] *Tgfb1* mRNA levels, significantly elevated at 3 days after laser injury, were reversed by intravitreal injection of (P)RR-PshRNA (Figure 3D). Given that RPE cells are the major source of TGF-β1,[34] we confirmed whether ERK1/2 signaling is required for (P)RR-mediated TGF-β1 expression *in vitro*. Prorenin stimulation to RPE cells significantly elevated the phosphorylation of ERK1/2 and the upregulation of TGF-β1, both of which were significantly abolished by pretreatment with the ERK1/2 inhibitor (U0126) and (P)RR-PshRNA (Figures 3E and 3F). At 3 days after laser injury, *Acta2*, *Col1a1*, and *Fn1* mRNA levels increased; however, these changes were also significantly attenuated by intravitreal injection of (P)RR-PshRNA (Figures 3G–3I), consistent with our recent data on TGF-β1-induced EMT changes in this model.[34]

Moreover, we examined EMT-related key transcription factors *Snai1* (SNAIL), *Snai2* (SLUG), and *Twist1* (TWIST) expression levels after CNV induction. Consistent with our previous data that SNAIL, but not SLUG or TWIST, was expressed in AMD patient samples,[35] we found that the *Snai1*, but not *Snai2* or *Twist1*, mRNA was significantly upregulated in the PRE-choroid complex at 3 days after laser treatment (Figures 3J, S2A, and S2B). Importantly, the intravitreal injection of (P)RR-PshRNA inhibited the upregulation of *Snai1* mRNA level in CNV mice (Figure 3J). TGF-β signaling with activation (i.e., phosphorylation and nuclear translocation) of SMAD family proteins has been recognized as the major trigger of EMT in tissue remodeling and fibrosis.[31,33] We next examined the effect of (P)RR-PshRNA on the activation of SMAD2. The protein levels of phosphorylated SMAD2, enhanced by laser injury, were significantly suppressed by (P)RR-PshRNA (Figures 3K and 3L). These results suggested that (P)RR-PshRNA inhibited the development of subretinal fibrosis through ERK1/2-mediated TGF-β/SMAD2/SNAIL signaling.

### Blockade of Inflammatory Responses by (P)RR-PshRNA in TNF-α-Stimulated Endothelial Cells and RPE Cells

Previously, we and others reported that TNF-α and lipopolysaccharide (LPS) stimulation induced (P)RR activation in retinal vascular endothelial cells and alveolar epithelial cells, leading to the activation of RAPS and subsequent expression of inflammatory molecules in animals.[11,36,37] To confirm our *in vivo* data, we further investigated whether (P)RR-PshRNA suppresses the expression of inflammatory and profibrotic molecules in TNF-α-stimulated murine microvascular endothelial cells (b.End3) and human RPE cells. TNF-α stimulation to endothelial cells and RPE cells significantly increased the mRNA levels of *Ccl2/CCL2*, *Icam1/ICAM1*, *Il6/IL6*, and *Tnfa/TNFA*, as well as *(P)RR/ATP6AP2* (Figures 4A–4E). *Tgfb1/TGFB1* did not respond to TNF-α stimulation (Figure 4F), which is rather complemented by our present and recent data showing the RPE induction of TGF-β by prorenin/(P)RR/ERK1/2 signaling (Figures 3E

**Figure 2. Inhibition of CNV-Related Inflammatory Molecule Expression, Macrophage Infiltration, and ERK1/2 Activation by (P)RR-PshRNA**

(A–F) Gene expression levels of inflammatory molecules *(P)RR/Atp6ap2* (A), *Ccl2* (B), *Icam1* (C), *Il6* (D), *Tnfa* (E), and *Emr1* (F) in the RPE-choroid complex of untreated normal mice (control) and CNV mice treated with 100 pmol control-PshRNA or (P)RR-PshRNA. *p < 0.05, **p < 0.01 (n = 6–8). (G–L) Representative micrographs of F4/80-positive macrophages (red) (G and J) in CNV lesions (isolectin B4, green) (H and K) from CNV mice treated with 100 pmol control-PshRNA or (P)RR-PshRNA. (I and L) Merged images. Scale bar, 50 μm. (M) Quantification of CNV area-adjusted number of F4/80-positive macrophages [control-PshRNA = 10.83 ± 2.48/1,000 $\mu m^2$, (P)RR-PshRNA = 3.17 ± 0.75/1,000 $\mu m^2$]. **p < 0.01 (n = 4). (N) Immunoblotting analysis for phosphorylated and total ERK1/2 in the RPE-choroid complex of control and CNV mice treated with 100 pmol control-PshRNA or (P)RR-PshRNA at day 3 after laser injury. (O) Densitometry values of phosphorylated ERK1/2 normalized to total ERK1/2. **p < 0.01 (n = 4).

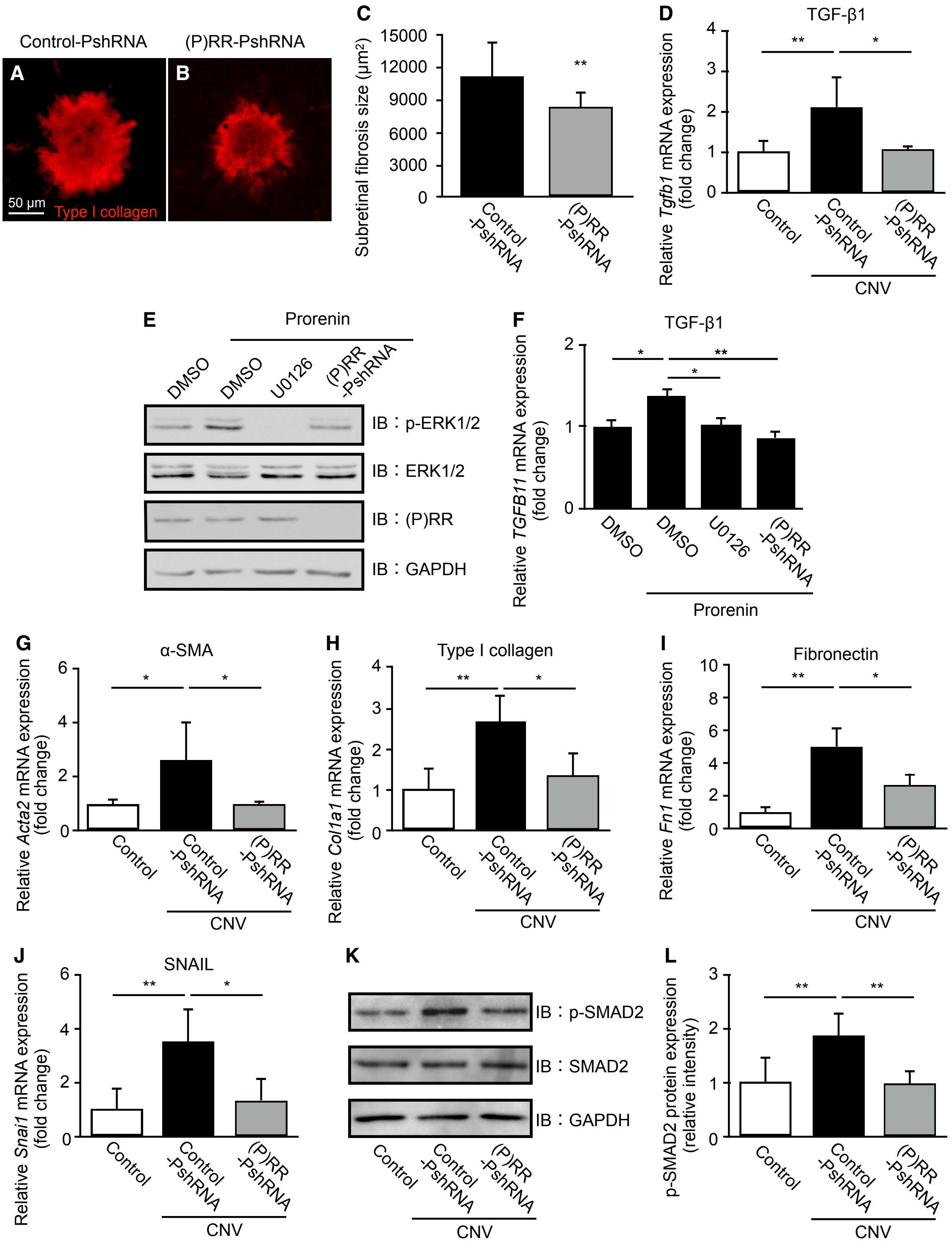


**Figure 3. Suppression of Subretinal Fibrosis and Fibrotic Molecules via the ERK1/2-Mediated TGF-β/SMAD2/SNAIL Pathway by (P)RR-PshRNA**

(A and B) Representative micrographs of subretinal fibrosis lesions (type I collagen, red) in the RPE-choroid flat mounts at post-laser day 7 from mice treated with 100 pmol control-PshRNA (A) or (P)RR-PshRNA (B). Scale bar, 50 μm. (C) Quantification analysis of the size of subretinal fibrosis [control-PshRNA = 11,149 ± 3,204 $\mu m^2$, (P)RR-PshRNA = 8,264 ± 1,376 $\mu m^2$]. **$p < 0.01$ (n = 6–8). (D) Gene expression level of profibrotic cytokine *Tgfb1* in the RPE-choroid complex of untreated normal mice (control) and CNV mice treated with 100 pmol control-PshRNA or (P)RR-PshRNA. *$p < 0.05$, **$p < 0.01$ (n = 6). (E) Immunoblotting for phosphorylated and total ERK1/2 and (P)RR in

*(legend continued on next page)*

and 3F) and by TGF-β/SMAD2 signaling (i.e., autoinduction).[34] Consistent with the *in vivo* results (Figures 2A–2E and 3D), the silencing of *(P)RR/ATP6AP2* by (P)RR-PshRNA prevented mRNA expression of these inflammatory and profibrotic molecules (Figures 4A–4F). Similarly, LPS-induced inflammatory responses in endothelial cells and RPE cells were also suppressed by (P)RR-PshRNA treatment (Figures S3A–S3F).

### Attenuation of CNV and Subretinal Fibrosis Formation in RPE-Specific *(P)RR/Atp6ap2*-CKO Mice

To further validate our findings of the anti-angiogenic and -fibrotic effects of (P)RR-PshRNA in mice (Figures 1 and 3), we generated RPE-specific *(P)RR/Atp6ap2*-conditional knockout (CKO) mice by crossing *loxP/Atp6ap2* mice[38,39] and *BEST1-Cre* transgenic mice. *BEST1-Cre* is expressed after post-natal day 10 in the RPE.[40] Preliminary results confirmed that *Atp6ap2* expression significantly decreased in the RPE-choroid complex of CKO mice compared with controls (Figures S4A and S4B). Next, we checked the impact of CNV induction on angiogenesis and fibrosis in *Atp6ap2*-CKO mice at 7 days after laser treatment. Both CNV and fibrosis sizes were significantly attenuated in *Atp6ap2*-CKO mice compared with those in controls (Figures 5A–5F).

### Comparison between (P)RR-PshRNA and Aflibercept in the Suppressive Effects of CNV and Subretinal Fibrosis Formation

Because (P)RR-PshRNA was shown to have potential as a possible candidate drug for the treatment of AMD (Figures 1, 2, and 3), we further compared the efficiency of (P)RR-PshRNA with that of aflibercept, one of the first-line anti-VEGF drugs in clinical practice,[41] for CNV and subretinal fibrosis formation. The sizes of CNV and subretinal fibrosis were quantified in CNV mice that received intravitreal injection of PBS, aflibercept at the clinically applied dose, (P)RR-PshRNA, and combination of aflibercept and (P)RR-PshRNA. CNV significantly decreased and subretinal fibrosis tended to decrease without statistical significance in mice treated with aflibercept (Figures 6A–6D, 6I, and 6J), whereas animals treated with (P) RR-PshRNA showed significant decreases in both CNV and subretinal fibrosis compared with those treated with PBS (Figures 6E, 6F, 6I, and 6J), in accordance with data shown in Figures 1 and 3. We also determined whether injection of a mixture of aflibercept and (P) RR-PshRNA achieves a larger inhibition than produced by each alone. Compared with PBS-treated animals, combination of aflibercept and (P)RR-PshRNA significantly decreased both CNV and subretinal fibrosis (Figures 6G–6J); however, we could detect no additive effect under this condition. These results suggested that the therapeutic effect of (P)RR-PshRNA was comparable with that of aflibercept.

### Co-localization of (P)RR with Phosphorylated ERK1/2 in AMD Patient Specimens

Previously, we demonstrated (P)RR localization in neovascular endothelial cells in the fibrovascular tissue from eyes with proliferative diabetic retinopathy and RPE cells of a postmortem human eye;[18,42] however, no data on AMD patient samples have been reported. To examine the tissue localization of (P)RR, we performed immunofluorescence for the CNV tissue in AMD patient specimens. Double-staining experiments demonstrated that (P)RR immunoreactivity was detected in CD34-positive neovascular endothelial cells (Figures 7A–7C) and RPE65-positive RPE cells (Figures 7G–7I), both of which showed co-localization with phosphorylated ERK1/2 (Figures 7D–7F and 7J–7L). (P)RR co-localization with phosphorylated ERK1/2-positive new vessels is comparable with our previous report on proliferative diabetic retinopathy.[18] These results indicated the involvement of (P)RR and its downstream ERK1/2 signaling activation in the pathogenesis of CNV and subretinal fibrosis in human AMD.

## DISCUSSION

The current study reveals, for the first time, several important findings concerning the involvement of (P)RR in the pathogenesis of CNV and subretinal fibrosis. Inhibition of (P)RR by intravitreal injection of (P) RR-PshRNA significantly suppressed CNV formation (Figure 1), the expression of CNV-related inflammatory molecules via the ERK1/2 pathway, and macrophage influx into the RPE-choroid complex in the murine model (Figure 2). Application of (P)RR-PshRNA suppressed the expression of *Tgfb1*, thereby suppressing subretinal fibrosis formation together with EMT-related markers including phosphorylated SMAD2 (Figure 3). Notably, prorenin stimulation to RPE cells significantly increased ERK1/2 phosphorylation, leading to upregulation of TGF-β1 expression (Figure 3). Consistent with the *in vivo* findings, (P)RR-PshRNA suppressed TNF-α-induced inflammatory and profibrotic molecules in endothelial cells and RPE cells (Figure 4). RPE-specific *(P)RR/Atp6ap2*-CKO mice exhibited amelioration of both CNV and subretinal fibrosis (Figure 5). In addition, the therapeutic effect of (P)RR-PshRNA was comparable with that of aflibercept (Figure 6). Importantly, AMD patient specimens demonstrated the co-localization of (P)RR with phosphorylated ERK1/2 in neovascular endothelial cells and RPE cells (Figure 7). These results suggest that the activation of (P)RR triggers molecular cascades for inflammation-related angiogenesis and EMT-driven fibrosis; consequently, intravitreal (P)RR-PshRNA injection to mice significantly inhibits the development of CNV and subretinal fibrosis (Figure 8).

A growing body of evidence has shown that tissue RAS and RAPS play several important roles in pathological vascular abnormalities, such as angiogenesis and inflammation, in various organs.[7–19,23,37]

human RPE cells stimulated with prorenin at 20 nM for 24 h following pretreatment with DMSO, and ERK1/2 inhibitor (U0126) at 10 μM for 30 min or (P)RR-PshRNA at 1 nM for 24 h. (F) Relative gene expression levels of *TGFB1* in prorenin-stimulated human RPE cells pretreated with DMSO, U0126, or (P)RR-PshRNA. $^{*}p < 0.05$, $^{**}p < 0.01$ (n = 6). (G–J) Gene expression level of EMT markers *Acta2* (G), *Col1a1* (H), *Fn1* (I), and *Snai1* (J) in the RPE-choroid complex of untreated normal mice (control) and CNV mice treated with 100 pmol control-PshRNA or (P)RR-PshRNA. $^{*}p < 0.05$, $^{**}p < 0.01$ (n = 6). (K) Immunoblotting for phosphorylated and total SMAD2 in the RPE-choroid complex of control and CNV mice treated with control-PshRNA or (P)RR-PshRNA at day 3 after laser photocoagulation. (L) Densitometry values of phosphorylated SMAD2 normalized to total SMAD2. $^{**}p < 0.01$ (n = 4).

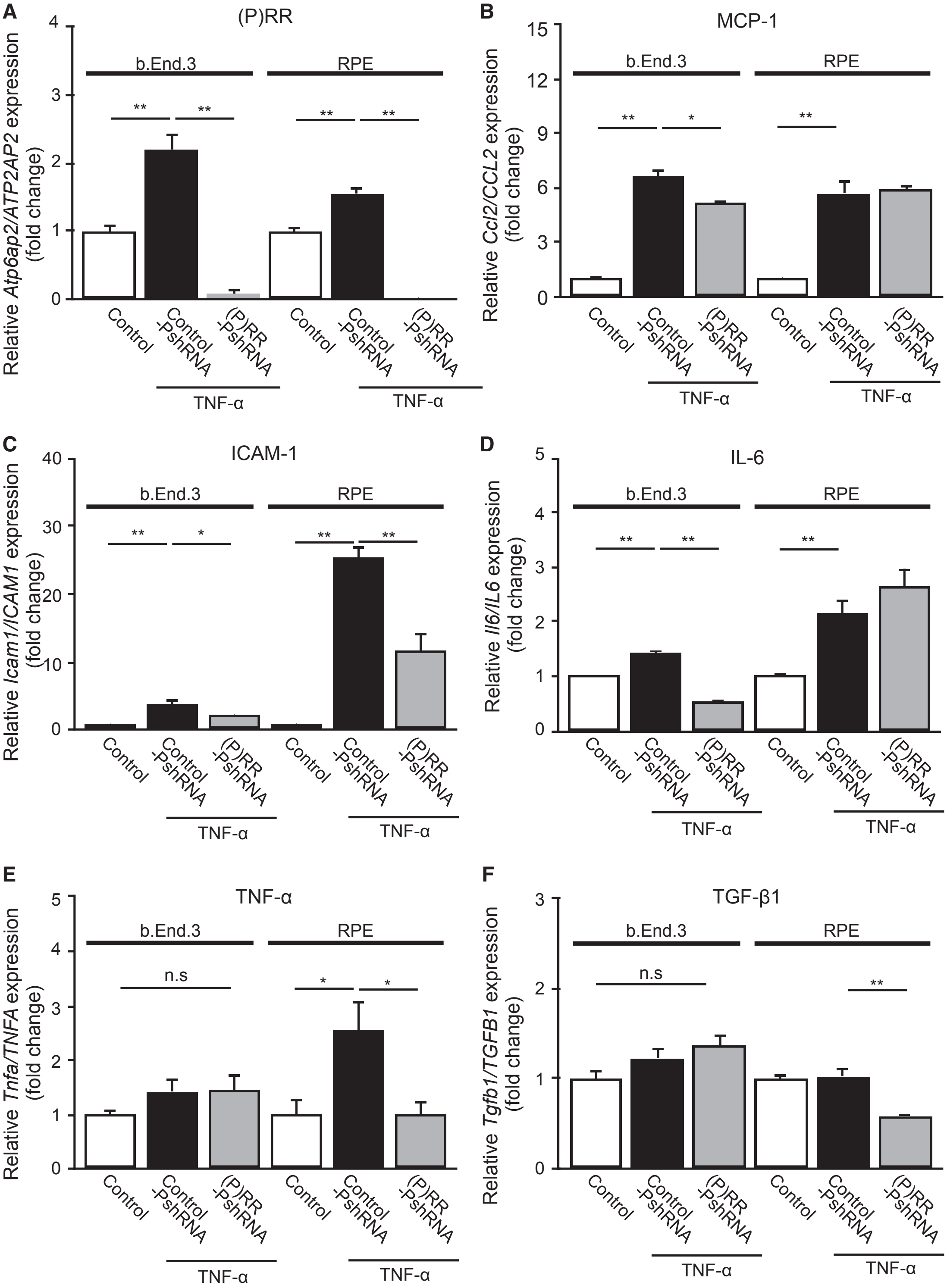


**Figure 4. Blockade of Inflammatory Responses by (P)RR-PshRNA in TNF-α-Stimulated Endothelial Cells and RPE Cells**

(A–F) Gene expression levels of inflammatory mediators *Atp6ap2/ATP6AP2* (A), *Ccl2/CCL2* (B), *Icam1/ICAM1* (C), *Il6/IL6* (D), *Tnfa/TNFA* (E), and profibrotic cytokine *Tgfb1/TGFB1* (F) in 1 nM control-PshRNA or (P)RR-PshRNA transfected murine microvascular endothelial cells stimulated with 2 ng/mL TNF-α for 3 h and 1 nM control-PshRNA or (P)RR-PshRNA transfected human RPE cells stimulated with 10 ng/mL TNF-α for 12 h. *p < 0.05, **p < 0.01 (n = 6). n.s., not significant.

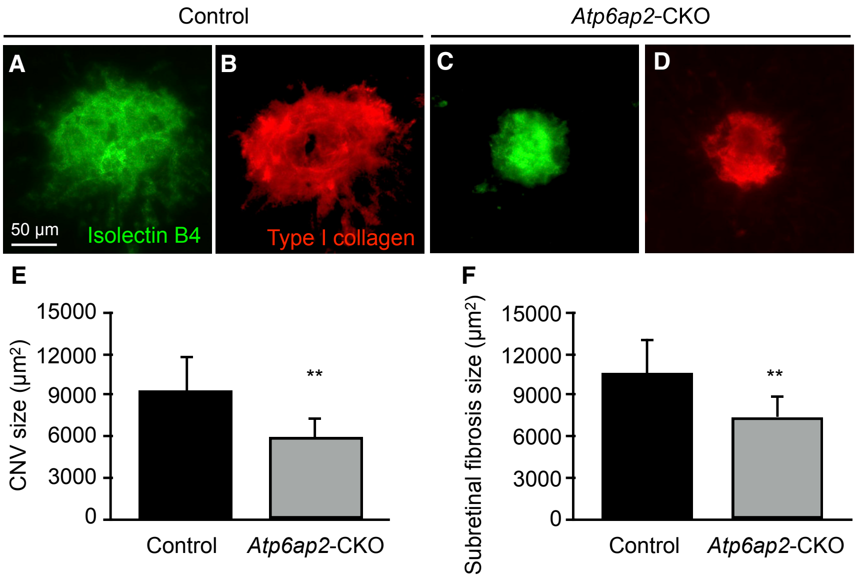


**Figure 5. Attenuation of CNV and Subretinal Fibrosis Formation in RPE-Specific *(P)RR/Atp6ap2* CKO Mice**

(A–D) Representative micrographs of CNV (isolectin B4, green) and subretinal fibrosis (type I collagen, red) in the RPE-choroid flat mount at day 7 after laser injury in control (A and B) and *(P)RR/Atp6ap2*-CKO (C and D) mice. Their littermates (e.g., *Atp6ap2*$^{lox/Y}$/*BEST1-Cre*$^{-/-}$) served as control mice. Scale bar, 50 μm. (E and F) Quantification analysis of the (E) size of CNV [control = 9,277 ± 2,590 $\mu m^2$, *(P)RR/Atp6ap2*-CKO = 5,963 ± 1,511 $\mu m^2$] and (F) subretinal fibrosis [control = 10,556 ± 3,172 $\mu m^2$, *(P)RR/Atp6ap2*-CKO = 7,452 ± 1,600 $\mu m^2$]. $^{**}p < 0.01$ (n = 6).

Inflammation plays important roles in the pathogenesis of CNV caused by endothelial production of CCL2/MCP-1,[24] ICAM-1,[25] IL-6,[26] and TNF-α,[27] all of which proved to be regulated by (P) RR,[9–12,23] as well as ERK1/2,[9,10,18] and responsible for the recruitment of proangiogenic macrophages.[25,29] Importantly, we previously reported that (P)RR co-localized with phosphorylated ERK1/2-expressing endothelial cells in the fibrovascular tissue surgically excised from human eyes with proliferative diabetic retinopathy,[18] and that RAS-independent intracellular signaling via (P)RR contributed to the activation of ERK1/2 in the retina of diabetic mice.[10] The current study is the first to show (P)RR expression in neovascular endothelial cells and its co-localization with phosphorylated ERK1/2 in the CNV tissue of AMD patient specimens (Figures 7A–7F). Inhibition of (P)RR using (P)RR-PshRNA significantly inhibited the inflammatory pathogenesis of CNV both *in vivo* and *in vitro* (Figures 1, 2, 4, and S3) followed by macrophage infiltration, consistent with our previous report.[9] Taken together, (P)RR activation would be a common mechanism for enhancing inflammation-related angiogenesis in the eye, which is driven in part by ERK1/2 phosphorylation and subsequent expression of inflammatory molecules.

Clinically, subretinal fibrosis has recently been regarded as a key pathological event in CNV, basically a form of fibrovascular proliferation consisting of both vascular and fibrous components, the latter of which would theoretically be and actually seemed to be resistant to anti-VEGF therapy.[5,6] As a major trigger for fibrotic changes, TGF-β1, a key multifunctional cytokine stimulated by ERK1/2 activation,[43] is known to induce the phosphorylation and translocation of SMAD proteins, leading to the differentiation of fibroblasts into myofibroblasts both *in vitro* and *in vivo*.[31–33] Indeed, AMD patient specimens previously demonstrated the potent immunoreactivity of TGF-β1 in CNV-associated RPE cells,[44] and the process of CNV-associated subretinal fibrosis was shown to be mainly caused by TGF-β1-SMAD2/3 signal transduction, leading to SNAIL-mediated EMT changes in RPE cells.[34,35,45,46] Our *in vivo* data showed that administration of (P)RR-PshRNA significantly suppressed CNV-related TGF-β1 expression, leading to reduction in phosphorylated SMAD2 and *Snai1* expression levels, thereby ameliorating subretinal fibrosis formation (Figure 3). Although RPE cells were shown to be equipped with an autoinduction system for TGF-β1 expression via TGF-β1/SMAD2 signaling,[34] little is known about upstream stimuli for triggering TGF-β1 upregulation in the first place during CNV-associated subretinal fibrosis formation. The current study is the first to show the contribution of the prorenin/(P)RR/ERK1/2 axis to the induction of TGF-β1 expression in RPE cells. Importantly, we revealed that (P)RR was expressed in RPE cells and co-localized with phosphorylated ERK1/2 in the CNV tissue of AMD patient specimens (Figures 7G–7L). Supporting our current results, previous studies using a genetic hypertension rat model and renal cell lines indicated that activation of (P)RR significantly increased the expression of TGF-β1, type I collagen, and fibronectin via the ERK1/2 pathway, all of which were abolished by (P)RR blockade,[14–17] suggesting the biological significance of (P)RR as a key promotor for fibrosis in various organs.

Intravitreal injection of VEGF blockers is a first-line therapy for several ocular disorders including AMD; however, the anti-VEGF strategy is not necessarily effective for all patients.[47,48] Despite the successful results of numerous prospective clinical trials, 10% to 30% of patients have been shown to be non-responders for anti-VEGF drugs. We revealed that the therapeutic effect of (P)RR-PshRNA was comparable with that of aflibercept, indicating its possibility of an alternative treatment option for AMD. In conclusion, our present data indicate that (P)RR plays pivotal roles in the pathogenesis of both inflammation-related angiogenesis and EMT-driven subretinal fibrosis in AMD, and also provide evidence that inhibition of (P)RR is a promising strategy in clinical practice.

## MATERIALS AND METHODS

### RNAi Agent Targeting *(P)RR/Atp6ap2*

A new class of single-stranded RNAi agent targeting both human and mouse *(P)RR/ATP6AP2*, (P)RR-PshRNA, was synthesized on a solid

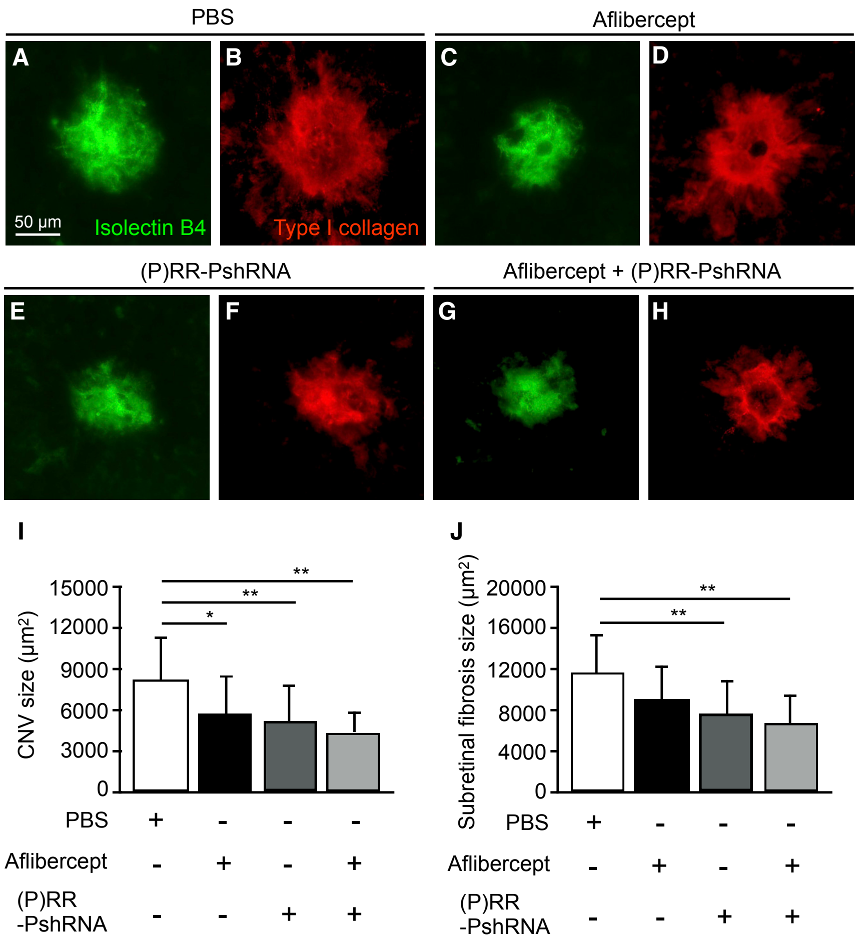


**Figure 6. Comparison of (P)RR-PshRNA and Aflibercept for Suppression of CNV and Subretinal Fibrosis Formation**

(A–H) Representative micrographs of CNV (isolectin B4, green) and subretinal fibrosis (type I collagen, red) in the RPE-choroid flat mount at day 7 after laser injury in mice treated with intravitreal injection of PBS (A and B), aflibercept (2.5 μg) (C and D), (P)RR-PshRNA (100 pmol) (E and F), and mixture of aflibercept and (P)RR-PshRNA (2.5 μg + 100 pmol) (G and H), respectively. Scale bar, 50 μm. (I and J) Quantification analysis of the (I) sizes of CNV [PBS = 8,253 ± 3,124 $\mu m^2$, aflibercept = 5,732 ± 2,795 $\mu m^2$, (P)RR-PshRNA = 5,224 ± 2,691 $\mu m^2$, aflibercept + (P)RR-PshRNA = 4,439 ± 1,535 $\mu m^2$] and (J) fibrosis [PBS = 11,746 ± 3,678 $\mu m^2$, aflibercept = 9,112 ± 3,322 $\mu m^2$, (P)RR-PshRNA = 7,610 ± 3,407 $\mu m^2$, aflibercept + (P)RR-PshRNA = 6,754 ± 2,895 $\mu m^2$]. *$p < 0.05$, **$p < 0.01$ (n = 5).

phase as a single-strand RNAi segment containing a short hairpin structure and used as follows: 5′-GUU UUC CGA AAU GGA AAU CC-P-GGA UUU CCA UUU CGG AAA ACA G-3′.[23] P indicates a proline derivative. A negative control-PshRNA was 5′-UAC UAU UCG ACA CGC GAA GUU CC-P-GGA ACU UCG CGU GUC GAA UAG UAU U-3′, as established previously.[23]

### Animals

C57BL/6J mice aged 6–8 weeks (CLEA Japan, Tokyo, Japan) were maintained in the animal facility at Hokkaido University. *Atp6ap2*-floxed mice[38,39] were bred with a *bestrophin 1 (BEST1)-Cre* transgenic mouse line (Jackson Laboratory, Bar Harbor, ME, USA), which express *Cre* recombinase specifically in the RPE.[40] All animal experiments were performed following the guidelines of the Association for Research in Vision and Ophthalmology (ARVO) Statement for the Use of Animals in Ophthalmic and Vision Research. The experimental protocols were approved by the Ethics Review Committee for Animal Experimentation of Hokkaido University.

### Laser-Induced CNV Model and Drug Application

CNVs were generated as described previously.[49] In brief, under deep anesthesia, laser photocoagulation (532 nm, 180 mW, 75 μm, 100 ms; Novus Spectra; Lumenis, Tokyo, Japan) was performed around the optic disc using a slit-lamp delivery system with a cover glass as a contact lens. Four laser spots per eye for immunohistochemistry and six laser spots for mRNA and protein analysis were placed. The formation of a subretinal bubble at the time of laser photocoagulation indicated the rupture of Bruch's membrane. (P)RR-PshRNA, aflibercept (2.5 μg; Bayer, Leverkusen, Germany), or a mixture of (P)RR-PshRNA and aflibercept in 1 μL PBS per eye was injected into the vitreous cavity of mice immediately after laser injury. An additional injection was given on day 7 for quantification of subretinal fibrosis at day 21 after laser. Controls received control-PshRNA or PBS. The dose of *in vivo* injection of control or (P)RR-PshRNA was determined to be 100 pM in 1 μL PBS per eye, at which (P)RR-PshRNA significantly suppresses the size of CNV in dose-ranging experiments.

### Measurement of CNV and Choroidal Fibrosis

Seven days after laser injury, eyes were enucleated and fixed in 4% paraformaldehyde. After removal of the anterior segment and retina, four radial incisions were made, and the remaining RPE-choroid complexes were incubated with isolectin B4-Alexa 488 (1:100; Thermo Fisher Scientific, Waltham, MA, USA) and rabbit anti-collagen type I antibody (1:100; Rockland Immunochemicals, Limerick, PA, USA) to detect CNV and choroidal fibrosis, respectively. The secondary antibody for type I collagen was Alexa 546 (1:200; Thermo Fisher Scientific). A fluorescence microscope (Biorevo; Keyence, Tokyo, Japan) was used to measure the area of CNV and choroidal fibrosis.

### RNA Isolation and Real-Time PCR Analyses

Total RNA isolation and reverse transcription were performed using TRIzol (Thermo Fisher Scientific) and GoScript Reverse

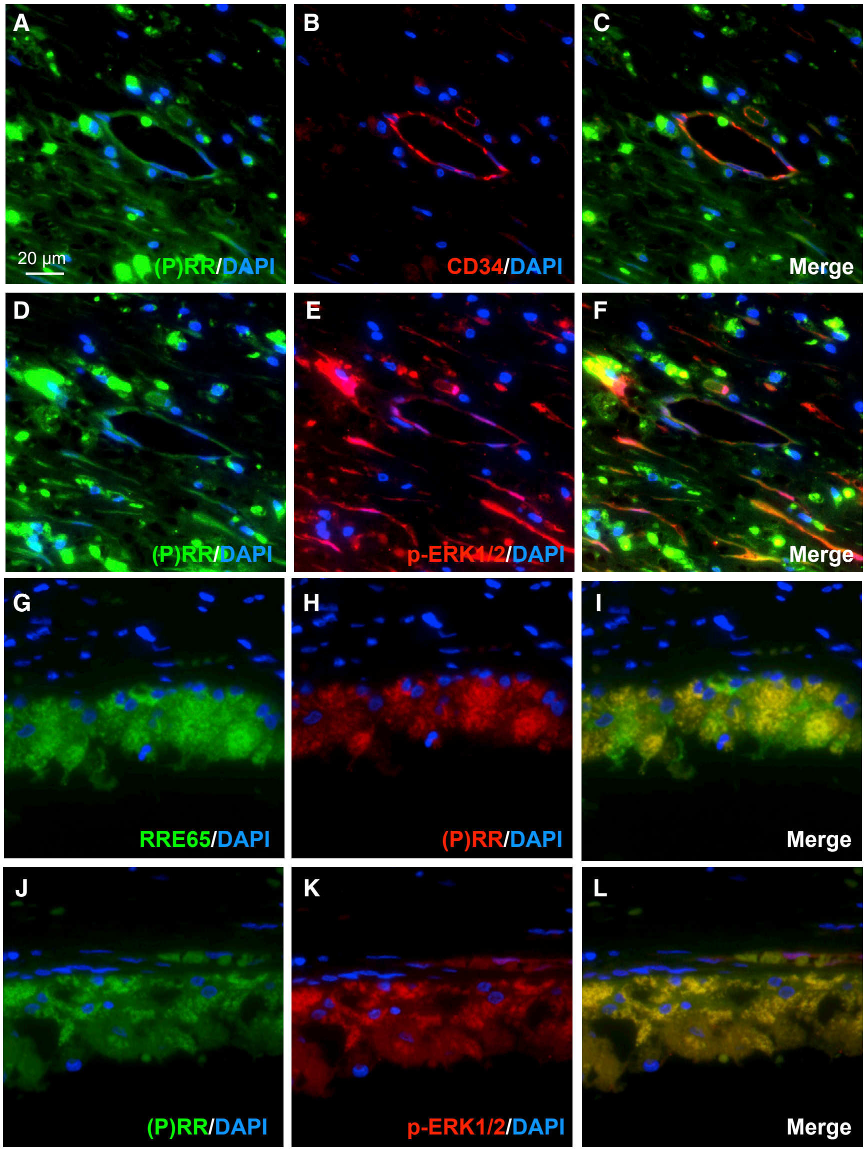


**Figure 7. Co-localization of (P)RR with Phosphorylated ERK1/2 in AMD Patient Specimens**

(A–L) Double labeling of (P)RR (green, A), CD34 (red, B), and DAPI (blue) (A–C); (P)RR (green, D), phosphorylated ERK1/2 (red, E), and DAPI (blue) (D–F); (P)RR (green, G), RPE65 (red, H), and DAPI (blue) (G–I); and (P)RR (green, J), phosphorylated ERK1/2 (red, K), and DAPI (blue) (J–L) in the CNV tissue specimens. (C, F, I, and L) Merged images. Scale bar, 20 μm.

Transcriptase (Promega, Madison, WI, USA) according to the manufacturer's protocols. All primers are listed in Table S1. Real-time qPCR was performed using the GoTaq qPCR Master mix (Promega) and StepOne Plus Systems (Thermo Fisher Scientific). Gene expression levels were calculated using the $2^{-ddCt}$ method, and all experimental samples were normalized using glyceraldehyde-3-phosphate dehydrogenase (*Gapdh*) as an internal control.

### Quantification of Infiltrating Macrophages

Three days after laser injury, the RPE-choroid complex was incubated with isolectin B4-Alexa 488 and rat anti-F4/80 antibody (1:100; Serotec, Oxford, UK). Alexa 546 goat anti-rat secondary antibody (1:200; Thermo Fisher Scientific) was then applied. The isolectin B4-stained area of CNV and F4/80-positive macrophages were quantified, and the area-adjusted number of macrophages was calculated.

### Immunoblot Analyses

The tissues and cells were lysed in SDS buffer. After quantifying protein concentrations using BCA reagent (Thermo Fisher Scientific), proteins (20 μg) were resolved by SDS-PAGE and transferred to the polyvinylidene difluoride (PVDF) membrane (Merck Millipore, Burlington, MA, USA) by electroblotting. Membranes were incubated with the following primary antibodies: rabbit anti-phosphorylated ERK1/2 (1:1,000), rabbit anti-ERK1/2 (1:1,000), rabbit anti-phosphorylated SMAD2 (1:1,000), rabbit anti-SMAD2 (1:1,000; Cell Signaling Technology, Danvers, MA, USA), rabbit anti-(P)RR antibody (1:1,000; Sigma-Aldrich), and mouse anti-GAPDH (1:2,000; Thermo Fisher Scientific). Horseradish peroxidase-conjugated anti-mouse and anti-rabbit IgGs (1:4,000; Jackson ImmunoResearch Laboratories, West Grove, PA, USA) were used as secondary antibodies for chemoluminescence detection. Signals were obtained by enhanced chemoluminescence (Western Lightning Ultra; Perkin Elmer, Waltham, MA, USA). The bands were analyzed by densitometry using ImageJ software (NIH, Bethesda, MD, USA).

### Cell Cultures and Transfection

Murine brain-derived capillary endothelial (b.End.3) and human RPE (hTERT-RPE1) cells were purchased from American Type Culture Collection (Manassas, VA, USA) and cultured in DMEM and DMEM/F12 (Fuji Film Wako Pure Chemicals, Osaka, Japan), respectively, supplemented with 10% fetal bovine serum (Thermo Fisher Scientific) at 37°C and 5% $CO_2$. Cells were transfected with RNAi agents using Lipofectamine RNAiMAX Reagent (Thermo Fisher Scientific) following the manufacturer's protocols. Twenty-four hours after transfection, the composite transfection mixture was removed and replaced with 1% FBS/DMEM for 24 h, followed with treatments before each assay. LPS and DMSO were

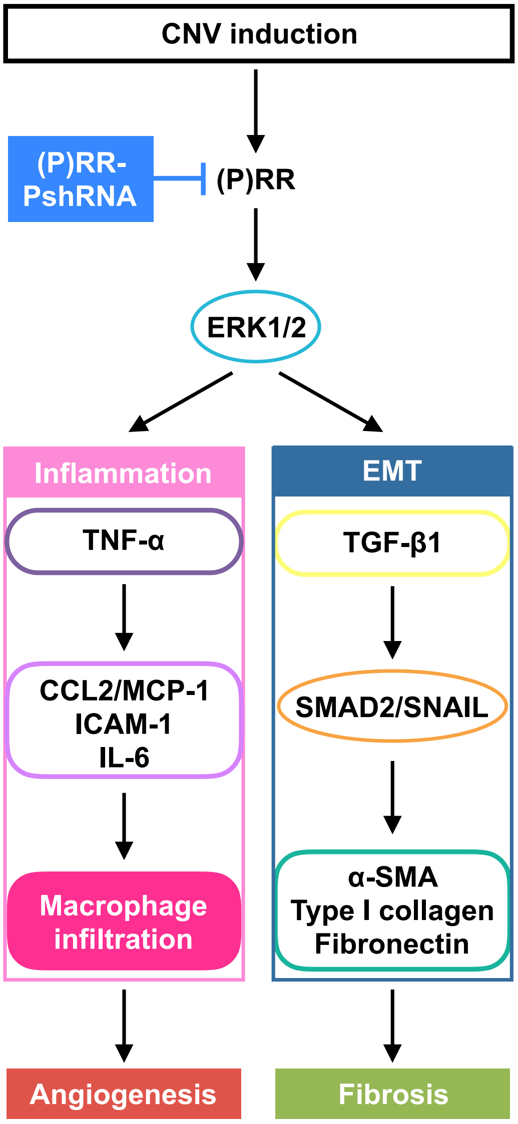


**Figure 8. A Schema Showing the Involvement of (P)RR with the Pathogenesis of CNV and Subretinal Fibrosis**

purchased from Sigma-Aldrich (St. Louis, MO, USA), human and mouse TNF-α recombinant protein from PeproTech (Rocky Hill, NJ, USA), human recombinant prorenin protein from Innovative Research (Novi, MI, USA), and ERK1/2 inhibitor (U0126) from Promega.

### Immunofluorescence Microscopy

AMD patient specimens were obtained in our clinic by enucleation due to suspected melanoma from an 82-year-old male with massive subretinal and vitreous hemorrhage secondary to CNV. This study was approved by the Ethics Committee of Hokkaido University Hospital, and written informed consent was obtained from the patient after an explanation of the purpose and consequence of this study. The enucleated globe was fixed with 4% paraformaldehyde and embedded with paraffin. Sections were deparaffinized and hydrated through exposure with xylene and graded alcohols followed by water. As a pretreatment, microwave-based antigen retrieval was performed in 10 mM citrate buffer (pH 6). These slides were incubated with the following primary antibodies: mouse anti-(P)RR (1:50),[18] rabbit anti-(P)RR (1:50; Sigma-Aldrich), rabbit anti-CD34 (1:100) and mouse anti-RPE65 (1:100; Abcam), and rabbit anti-phosphorylated ERK1/2 (1:100; Cell Signaling Technology) antibodies.

### Statistical Analysis

All the results are expressed as the mean ± SEM. Student's t test was used for statistical comparison between groups, and one-way ANOVA followed by the Tukey-Kramer method as a post hoc test was used for multiple comparison procedures. Differences between means were considered statistically significant when p values were <0.05.

## SUPPLEMENTAL INFORMATION

Supplemental Information can be found online at https://doi.org/10.1016/j.omtn.2019.05.012.

## AUTHOR CONTRIBUTIONS

A.K. designed the study; Y.L., A.K., D.W., E.T.I., S.K., K.N., and A.I. performed the experiments; Y.L. and A.K. analyzed the data; and Y.L., A.K., and S.I. wrote the paper. All authors approved the final version submitted for publication.

## CONFLICTS OF INTEREST

The patent on (pro)renin receptor (WPO Patent WO/2017/115872) became public, and in this patent, the names of A.K. and S.I. are included. The other authors declare no competing interests.

## ACKNOWLEDGMENTS

We thank Ikuyo Hirose, Shiho Yoshida, and Miyuki Murata (Hokkaido University) for their skilled technical assistance. This work was supported in part by the Bayer Japan Retina Award (to A.K.), the Institute of Science of Blood Pressure and Hormone (to A.K.), the New Energy and Industrial Technology Development Organization (NEDO), and MEXT KAKENHI grant numbers 16K11279 (to A.K.) and 16H05484 (to S.I.). Y.L. and D.W. are recipients of a scholarship from the China Scholarship Council (201508210220) and Otsuka Toshimi Scholarship Foundation, respectively.

## REFERENCES

1. de Jong, P.T.V.M. (2006). Age-related macular degeneration. N. Engl. J. Med. *355*, 1474–1485.
2. Willoughby, A.S., Ying, G.S., Toth, C.A., Maguire, M.G., Burns, R.E., Grunwald, J.E., Daniel, E., and Jaffe, G.J.; Comparison of Age-Related Macular Degeneration Treatments Trials Research Group (2015). Subretinal Hyperreflective Material in the Comparison of Age-Related Macular Degeneration Treatments Trials. Ophthalmology *122*, 1846–1853.e5.
3. Friedlander, M. (2007). Fibrosis and diseases of the eye. J. Clin. Invest. *117*, 576–586.
4. Miller, J.W., Le Couter, J., Strauss, E.C., and Ferrara, N. (2013). Vascular endothelial growth factor a in intraocular vascular disease. Ophthalmology *120*, 106–114.

5. Bloch, S.B., Lund-Andersen, H., Sander, B., and Larsen, M. (2013). Subfoveal fibrosis in eyes with neovascular age-related macular degeneration treated with intravitreal ranibizumab. Am. J. Ophthalmol. *156*, 116–124.e1.

6. Daniel, E., Toth, C.A., Grunwald, J.E., Jaffe, G.J., Martin, D.F., Fine, S.L., Huang, J., Ying, G.S., Hagstrom, S.A., Winter, K., and Maguire, M.G.; Comparison of Age-related Macular Degeneration Treatments Trials Research Group (2014). Risk of scar in the comparison of age-related macular degeneration treatments trials. Ophthalmology *121*, 656–666.

7. Nagai, N., Oike, Y., Noda, K., Urano, T., Kubota, Y., Ozawa, Y., Shinoda, H., Koto, T., Shinoda, K., Inoue, M., et al. (2005). Suppression of ocular inflammation in endotoxin-induced uveitis by blocking the angiotensin II type 1 receptor. Invest. Ophthalmol. Vis. Sci. *46*, 2925–2931.

8. Nagai, N., Oike, Y., Izumi-Nagai, K., Urano, T., Kubota, Y., Noda, K., Ozawa, Y., Inoue, M., Tsubota, K., Suda, T., and Ishida, S. (2006). Angiotensin II type 1 receptor-mediated inflammation is required for choroidal neovascularization. Arterioscler. Thromb. Vasc. Biol. *26*, 2252–2259.

9. Satofuka, S., Ichihara, A., Nagai, N., Noda, K., Ozawa, Y., Fukamizu, A., Tsubota, K., Itoh, H., Oike, Y., and Ishida, S. (2008). (Pro)renin receptor promotes choroidal neovascularization by activating its signal transduction and tissue renin-angiotensin system. Am. J. Pathol. *173*, 1911–1918.

10. Satofuka, S., Ichihara, A., Nagai, N., Noda, K., Ozawa, Y., Fukamizu, A., Tsubota, K., Itoh, H., Oike, Y., and Ishida, S. (2009). (Pro)renin receptor-mediated signal transduction and tissue renin-angiotensin system contribute to diabetes-induced retinal inflammation. Diabetes *58*, 1625–1633.

11. Satofuka, S., Ichihara, A., Nagai, N., Yamashiro, K., Koto, T., Shinoda, H., Noda, K., Ozawa, Y., Inoue, M., Tsubota, K., et al. (2006). Suppression of ocular inflammation in endotoxin-induced uveitis by inhibiting nonproteolytic activation of prorenin. Invest. Ophthalmol. Vis. Sci. *47*, 2686–2692.

12. Satofuka, S., Ichihara, A., Nagai, N., Koto, T., Shinoda, H., Noda, K., Ozawa, Y., Inoue, M., Tsubota, K., Itoh, H., et al. (2007). Role of nonproteolytically activated prorenin in pathologic, but not physiologic, retinal neovascularization. Invest. Ophthalmol. Vis. Sci. *48*, 422–429.

13. Ichihara, A., Hayashi, M., Kaneshiro, Y., Suzuki, F., Nakagawa, T., Tada, Y., Koura, Y., Nishiyama, A., Okada, H., Uddin, M.N., et al. (2004). Inhibition of diabetic nephropathy by a decoy peptide corresponding to the "handle" region for nonproteolytic activation of prorenin. J. Clin. Invest. *114*, 1128–1135.

14. Ichihara, A., Kaneshiro, Y., Takemitsu, T., Sakoda, M., Suzuki, F., Nakagawa, T., Nishiyama, A., Inagami, T., and Hayashi, M. (2006). Nonproteolytic activation of prorenin contributes to development of cardiac fibrosis in genetic hypertension. Hypertension *47*, 894–900.

15. Huang, Y., Wongamorntham, S., Kasting, J., McQuillan, D., Owens, R.T., Yu, L., Noble, N.A., and Border, W. (2006). Renin increases mesangial cell transforming growth factor-β1 and matrix proteins through receptor-mediated, angiotensin II-independent mechanisms. Kidney Int. *69*, 105–113.

16. Narumi, K., Sato, E., Hirose, T., Yamamoto, T., Nakamichi, T., Miyazaki, M., Sato, H., and Ito, S. (2018). (Pro)renin receptor is involved in mesangial fibrosis and matrix expansion. Sci. Rep. *8*, 16.

17. He, M., Zhang, L., Shao, Y., Wang, X., Huang, Y., Yao, T., and Lu, L. (2009). Inhibition of renin/prorenin receptor attenuated mesangial cell proliferation and reduced associated fibrotic factor release. Eur. J. Pharmacol. *606*, 155–161.

18. Kanda, A., Noda, K., Saito, W., and Ishida, S. (2012). (Pro)renin receptor is associated with angiogenic activity in proliferative diabetic retinopathy. Diabetologia *55*, 3104–3113.

19. Dong, Y., Kanda, A., Noda, K., Saito, W., and Ishida, S. (2017). Pathologic roles of receptor-associated Prorenin system in idiopathic Epiretinal membrane. Sci. Rep. *7*, 44266.

20. Biswas, K.B., Nabi, A.H., Arai, Y., Nakagawa, T., Ebihara, A., Ichihara, A., Watanabe, T., Inagami, T., and Suzuki, F. (2010). Aliskiren binds to renin and prorenin bound to (pro)renin receptor in vitro. Hypertens. Res. *33*, 1053–1059.

21. Suzuki, F., Hayakawa, M., Nakagawa, T., Nasir, U.M., Ebihara, A., Iwasawa, A., Ishida, Y., Nakamura, Y., and Murakami, K. (2003). Human prorenin has "gate and handle" regions for its non-proteolytic activation. J. Biol. Chem. *278*, 22217–22222.

22. Kleinman, M.E., Yamada, K., Takeda, A., Chandrasekaran, V., Nozaki, M., Baffi, J.Z., Albuquerque, R.J., Yamasaki, S., Itaya, M., Pan, Y., et al. (2008). Sequence- and target-independent angiogenesis suppression by siRNA via TLR3. Nature *452*, 591–597.

23. Kanda, A., Ishizuka, E.T., Shibata, A., Matsumoto, T., Toyofuku, H., Noda, K., Namba, K., and Ishida, S. (2017). A Novel Single-Strand RNAi Therapeutic Agent Targeting the (Pro)renin Receptor Suppresses Ocular Inflammation. Mol. Ther. Nucleic Acids *7*, 116–126.

24. Tsutsumi, C., Sonoda, K.H., Egashira, K., Qiao, H., Hisatomi, T., Nakao, S., Ishibashi, M., Charo, I.F., Sakamoto, T., Murata, T., and Ishibashi, T. (2003). The critical role of ocular-infiltrating macrophages in the development of choroidal neovascularization. J. Leukoc. Biol. *74*, 25–32.

25. Sakurai, E., Taguchi, H., Anand, A., Ambati, B.K., Gragoudas, E.S., Miller, J.W., Adamis, A.P., and Ambati, J. (2003). Targeted disruption of the CD18 or ICAM-1 gene inhibits choroidal neovascularization. Invest. Ophthalmol. Vis. Sci. *44*, 2743–2749.

26. Izumi-Nagai, K., Nagai, N., Ozawa, Y., Mihara, M., Ohsugi, Y., Kurihara, T., Koto, T., Satofuka, S., Inoue, M., Tsubota, K., et al. (2007). Interleukin-6 receptor-mediated activation of signal transducer and activator of transcription-3 (STAT3) promotes choroidal neovascularization. Am. J. Pathol. *170*, 2149–2158.

27. Wang, H., Han, X., Wittchen, E.S., and Hartnett, M.E. (2016). TNF-α mediates choroidal neovascularization by upregulating VEGF expression in RPE through ROS-dependent β-catenin activation. Mol. Vis. *22*, 116–128.

28. Sakurai, E., Anand, A., Ambati, B.K., van Rooijen, N., and Ambati, J. (2003). Macrophage depletion inhibits experimental choroidal neovascularization. Invest. Ophthalmol. Vis. Sci. *44*, 3578–3585.

29. Ishida, S., Usui, T., Yamashiro, K., Kaji, Y., Amano, S., Ogura, Y., Hida, T., Oguchi, Y., Ambati, J., Miller, J.W., et al. (2003). VEGF164-mediated inflammation is required for pathological, but not physiological, ischemia-induced retinal neovascularization. J. Exp. Med. *198*, 483–489.

30. Austyn, J.M., and Gordon, S. (1981). F4/80, a monoclonal antibody directed specifically against the mouse macrophage. Eur. J. Immunol. *11*, 805–815.

31. Kalluri, R., and Weinberg, R.A. (2009). The basics of epithelial-mesenchymal transition. J. Clin. Invest. *119*, 1420–1428.

32. Thiery, J.P., and Sleeman, J.P. (2006). Complex networks orchestrate epithelial-mesenchymal transitions. Nat. Rev. Mol. Cell Biol. *7*, 131–142.

33. Lamouille, S., Xu, J., and Derynck, R. (2014). Molecular mechanisms of epithelial-mesenchymal transition. Nat. Rev. Mol. Cell Biol. *15*, 178–196.

34. Wu, D., Kanda, A., Liu, Y., Kase, S., Noda, K., and Ishida, S. (2019). Galectin-1 promotes choroidal neovascularization and subretinal fibrosis mediated via epithelial-mesenchymal transition. FASEB J. *33*, 2498–2513.

35. Hirasawa, M., Noda, K., Noda, S., Suzuki, M., Ozawa, Y., Shinoda, K., Inoue, M., Ogawa, Y., Tsubota, K., and Ishida, S. (2011). Transcriptional factors associated with epithelial-mesenchymal transition in choroidal neovascularization. Mol. Vis. *17*, 1222–1230.

36. Ishii, K., Takeuchi, H., Fukunaga, K., Hirano, Y., Suda, K., Hagiwara, T., Miyasho, T., Yamada, S., Nakamura, R., Takahashi, T., et al. (2015). Attenuation of lipopolysaccharide-induced acute lung injury after (pro)renin receptor blockade. Exp. Lung Res. *41*, 199–207.

37. Hase, K., Kanda, A., Hirose, I., Noda, K., and Ishida, S. (2017). Systemic factors related to soluble (pro)renin receptor in plasma of patients with proliferative diabetic retinopathy. PLoS ONE *12*, e0189696.

38. Kinouchi, K., Ichihara, A., Sano, M., Sun-Wada, G.H., Wada, Y., Kurauchi-Mito, A., Bokuda, K., Narita, T., Oshima, Y., Sakoda, M., et al. (2010). The (pro)renin receptor/ATP6AP2 is essential for vacuolar H+-ATPase assembly in murine cardiomyocytes. Circ. Res. *107*, 30–34.

39. Kanda, A., Noda, K., Yuki, K., Ozawa, Y., Furukawa, T., Ichihara, A., and Ishida, S. (2013). Atp6ap2/(pro)renin receptor interacts with Par3 as a cell polarity determinant required for laminar formation during retinal development in mice. J. Neurosci. *33*, 19341–19351.

40. Iacovelli, J., Zhao, C., Wolkow, N., Veldman, P., Gollomp, K., Ojha, P., Lukinova, N., King, A., Feiner, L., Esumi, N., et al. (2011). Generation of Cre transgenic mice with

postnatal RPE-specific ocular expression. Invest. Ophthalmol. Vis. Sci. *52*, 1378–1383.

41. Heier, J.S., Brown, D.M., Chong, V., Korobelnik, J.-F., Kaiser, P.K., Nguyen, Q.D., Kirchhof, B., Ho, A., Ogura, Y., Yancopoulos, G.D., et al.; VIEW 1 and VIEW 2 Study Groups (2012). Intravitreal aflibercept (VEGF trap-eye) in wet age-related macular degeneration. Ophthalmology *119*, 2537–2548.

42. Kanda, A., Noda, K., and Ishida, S. (2015). ATP6AP2/(pro)renin receptor contributes to glucose metabolism via stabilizing the pyruvate dehydrogenase E1 β subunit. J. Biol. Chem. *290*, 9690–9700.

43. Fujita, H., Omori, S., Ishikura, K., Hida, M., and Awazu, M. (2004). ERK and p38 mediate high-glucose-induced hypertrophy and TGF-beta expression in renal tubular cells. Am. J. Physiol. Renal Physiol. *286*, F120–F126.

44. Amin, R., Puklin, J.E., and Frank, R.N. (1994). Growth factor localization in choroidal neovascular membranes of age-related macular degeneration. Invest. Ophthalmol. Vis. Sci. *35*, 3178–3188.

45. Ishikawa, K., Kannan, R., and Hinton, D.R. (2016). Molecular mechanisms of subretinal fibrosis in age-related macular degeneration. Exp. Eye Res. *142*, 19–25.

46. Iwanishi, H., Fujita, N., Tomoyose, K., Okada, Y., Yamanaka, O., Flanders, K.C., and Saika, S. (2016). Inhibition of development of laser-induced choroidal neovascularization with suppression of infiltration of macrophages in Smad3-null mice. Lab. Invest. *96*, 641–651.

47. Otsuji, T., Nagai, Y., Sho, K., Tsumura, A., Koike, N., Tsuda, M., Nishimura, T., and Takahashi, K. (2013). Initial non-responders to ranibizumab in the treatment of age-related macular degeneration (AMD). Clin. Ophthalmol. *7*, 1487–1490.

48. Nagai, N., Suzuki, M., Uchida, A., Kurihara, T., Kamoshita, M., Minami, S., Shinoda, H., Tsubota, K., and Ozawa, Y. (2016). Non-responsiveness to intravitreal aflibercept treatment in neovascular age-related macular degeneration: implications of serous pigment epithelial detachment. Sci. Rep. *6*, 29619.

49. Kanda, A., Noda, K., Saito, W., and Ishida, S. (2015). Aflibercept Traps Galectin-1, an Angiogenic Factor Associated with Diabetic Retinopathy. Sci. Rep. *5*, 17946.

**OMTN, Volume *17***

## Supplemental Information

# Suppression of Choroidal Neovascularization and Fibrosis by a Novel RNAi Therapeutic Agent against (Pro)renin Receptor

**Ye Liu, Atsuhiro Kanda, Di Wu, Erdal Tan Ishizuka, Satoru Kase, Kousuke Noda, Atsuhiro Ichihara, and Susumu Ishida**

# Supplemental information

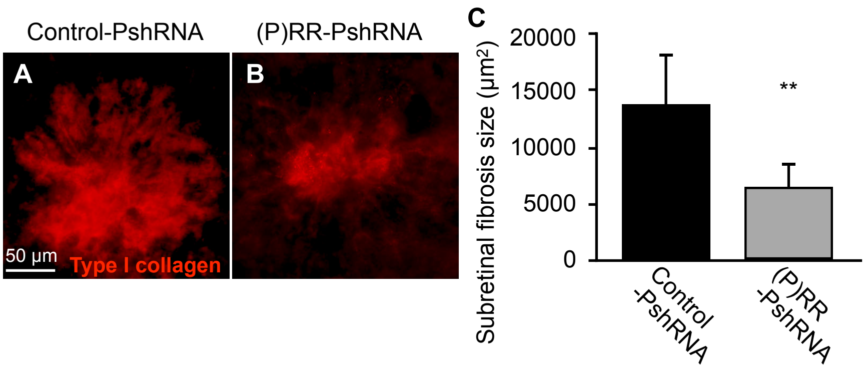


**Figure S1. Suppression of subretinal fibrosis at 21 days after laser photocoagulation by (P)RR-PshRNA**

(A, B) Representative micrographs of subretinal fibrosis lesions (type I collagen, *red*) in the RPE-choroid flat mounts at post-laser day 21 from mice treated with 100 pmol control-PshRNA (A) or (P)RR-PshRNA (B). Scale bar, 50 μm. (C) Quantification analysis of the size of subretinal fibrosis [control-PshRNA = 13,695 ± 4,566 $\mu m^2$, (P)RR-PshRNA = 6,381 ± 2,236 $\mu m^2$]. $^{**}p < 0.01$ (n = 5).

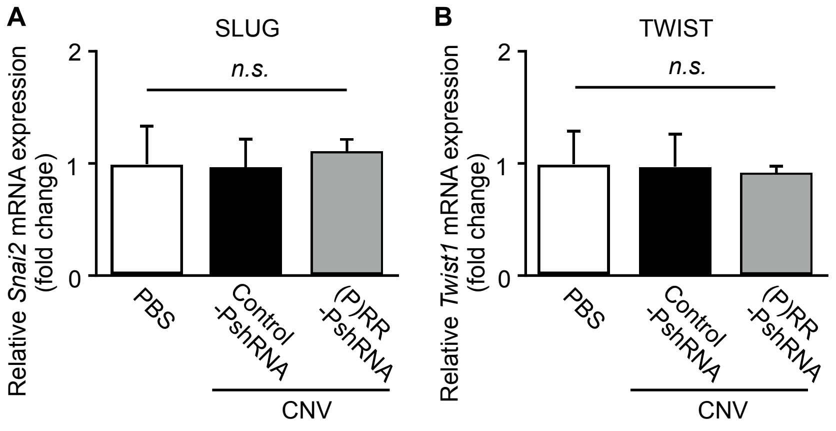


**Figure S2. Neither SNAI2 nor TWIST1 is associated with CNV induction in mice**

(A, B) Relative mRNA expression levels of *Snai2* (A) and *Twist1* (B) in the RPE-choroid complex from untreated normal mice (control) and CNV mice treated with 100 pmol control-PshRNA or (P)RR-PshRNA. n.s., not significant (n = 6).

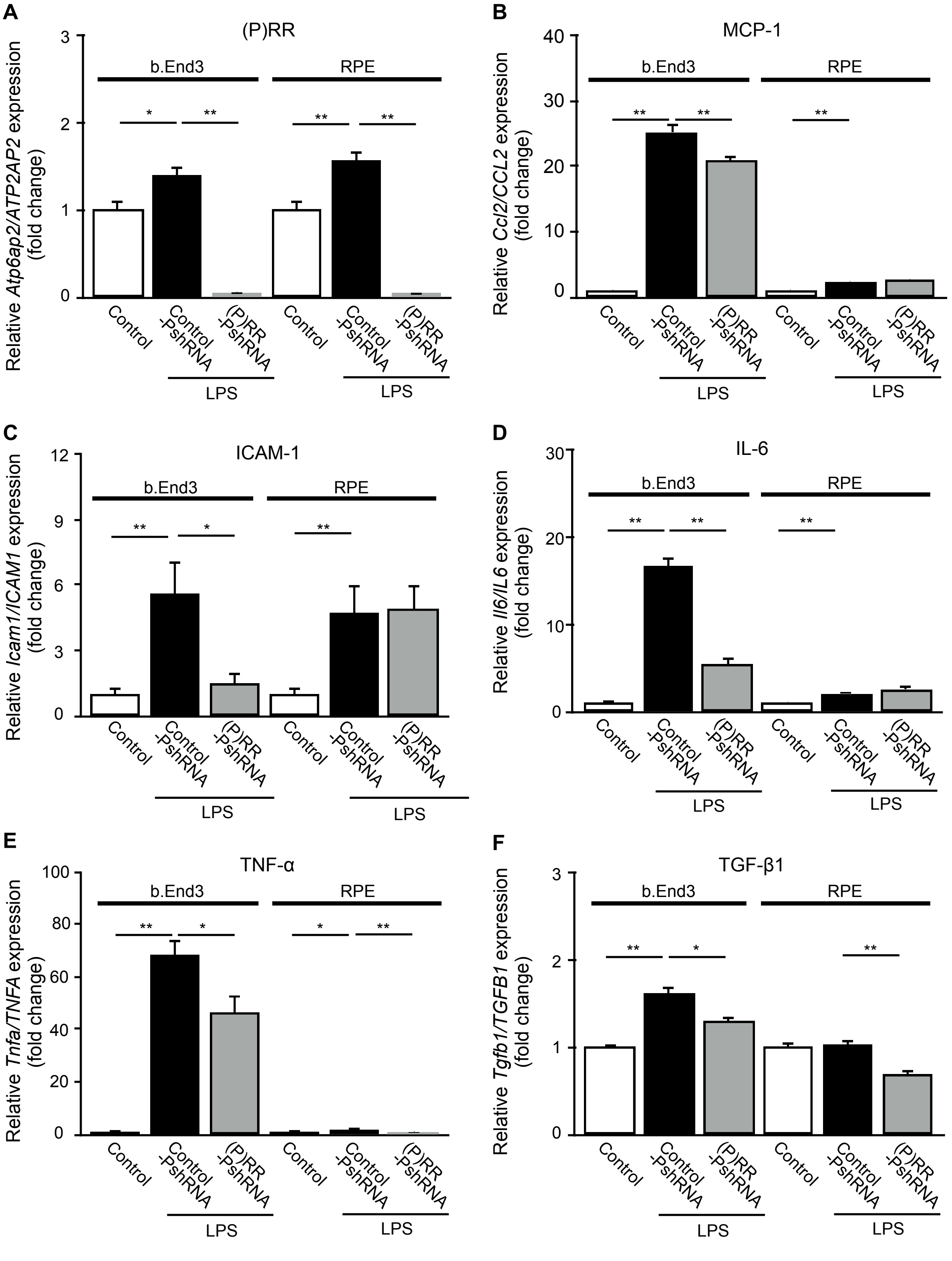


**Figure S3. Blockade of inflammatory responses by (P)RR-PshRNA in LPS-stimulated endothelial cells and RPE cells**

(A-E) Gene expression levels of inflammatory molecules *Atp6ap2/ATP6AP2* (A), *Ccl2/CCL2* (B), *Icam1/ICAM1* (C), *Il6/IL6* (D), *Tnfa/TNFA* (E) and profibrotic cytokine *Tgfb1/TGFB1* (F) in 1 nM control-PshRNA or (P)RR-PshRNA transfected mouse microvascular endothelial cells stimulated with 10 ng/ml LPS for 8 hours and 1 nM control-PshRNA or (P)RR-PshRNA transfected human RPE cells stimulated with 10 ng/ml LPS for 12 hours. $*p < 0.05$, $**p < 0.01$ (n = 6).

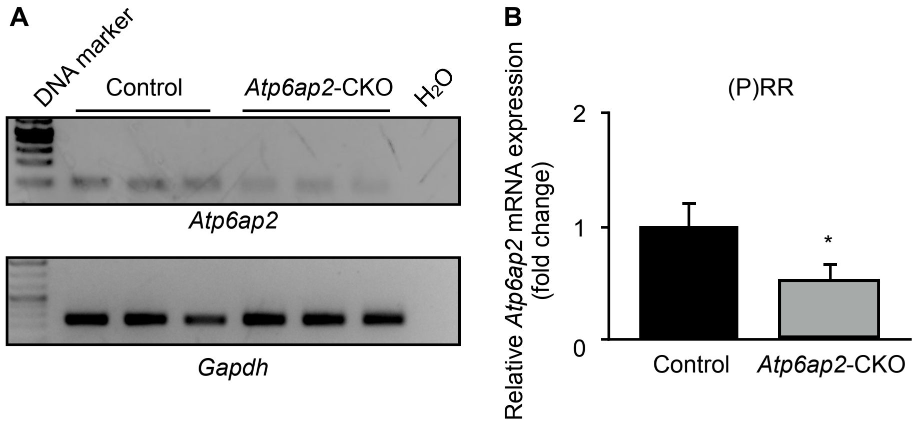


**Figure S4. Gene expression of *(P)RR/Atp6ap2* in the RPE-choroid complex of *(P)RR*/*Atp6ap2*-CKO mice**

(A) Gene expression analysis of *(P)RR/Atp6ap2* in the RPE-choroid complex of control and *(P)RR*/ *Atp6ap2*-CKO mice. *Gapdh* was used as an internal control. (B) Quantification analysis of the relative expression of *(P)RR*/*Atp6ap2*. *$p < 0.05$ (n = 3).

**Table S1. Primer sequences used in quantitative RT-PCR**

| Target gene | Sequence |
|---|---|
| Mouse | |
| *(P)RR/Atp6ap2* | forward 5'- CCTCATTAGGAAGACAAGGACTATCC -3' |
| | reverse 5'- GGGTTCTTCGCTTGTTTTGC -3' |
| *Ccl2* | forward 5'- TTGGCTCAGCCAGATGCA -3' |
| | reverse 5'- CCTACTCATTGGGATCATCTTGC -3' |
| *Icam1* | forward 5'- CCTGTTTCCTGGCTCTGAAG -3' |
| | reverse 5'- GTCTGCTGAGACCCCTCTTG -3' |
| *Il6* | forward 5'- CACAGAGGATACCACTCCCAACA -3' |
| | reverse 5'- TCCACGATTTCCCAGAGAAACA -3' |
| *Tnfa* | forward 5'- GGTGCCTATGTCTCAGCCTCTT -3' |
| | reverse 5'- CGATCACCCCGAAGTTCAGTA -3' |
| *Emr1* | forward 5'- CTTTGGCTATGGGCTTCCAGTC -3' |
| | reverse 5'- GCAAGGAGGACAGAGTTTATCGTG -3' |
| *Tgfb1* | forward 5'- CAGTGGCTGAACCAAGGAGAC -3' |
| | reverse 5'- ATCCCGTTGATTTCCACGTG -3' |
| *Acta2* | forward 5'- TCTGTAAGGCCGGCTTTGC -3' |
| | reverse 5'- TGTCCCATTCCCACCATCA -3' |
| *Col1a1* | forward 5'- TGACTGGAAGAGCGGAGAGT -3' |
| | reverse 5'- GACGGCTGAGTAGGGAACAC -3' |
| *Fn1* | forward 5'- GTCAGTGTCTCCAGTGTCTAC -3' |
| | reverse 5'- TGGCTTGCTGGCCAATCAGT -3' |
| *Snai1* | forward 5'- CACACGCTGCCTTGTGTCT -3' |
| | reverse 5'- GGTCAGCAAAAGCACGGTT -3' |
| *Snai2* | forward 5'- CAGCGAACTGGACACACACA -3' |
| | reverse 5'- ATAGGGCTGTATGCTCCCGAG -3' |
| *Twist1* | forward 5'- GGACAAGCTGAGCAAGATTCA -3' |
| | reverse 5'- CGGAGAAGGCGTAGCTGAG -3' |
| *Gapdh* | forward 5'- AGGTCGGTGTGAACGGATTTG -3' |
| | reverse 5'- TGTAGACCATGTAGTTGAGGTCA -3' |
| Human | |
| *(P)RR/ATP6AP2* | forward 5'- AGGCAGTGTCATTTCGTACC -3' |
| | reverse 5'- GCCTTCCCTACCATATACACTC -3' |
| *CCL2* | forward 5'- CGCCTCCAGCATGAAAGTCT -3' |
| | reverse 5'- ATGAAGGTGGCTGCTATG -3' |
| *ICAM1* | forward 5'- GCAAGCTCCCAGTGAAATGCAAAC -3' |
| | reverse 5'- TGTCTACTGACCCCAACCCTTGATG -3' |
| *IL6* | forward 5'- CCACTCACCTCTTCAGAACG -3' |
| | reverse 5'- CATCTTTGGAAGGTTCAGGTTG -3' |
| *TNFA* | forward 5'- ACTTTGGAGTGATCGGCC -3' |
| | reverse 5'- GCTTGAGGGTTTGCTACAAC -3' |
| *TGFB1* | forward 5'- GCCCTGGACACCAACTATTG -3' |
| | reverse 5'- CGTGTCCAGGCTCCAAATG -3' |
| *GAPDH* | forward 5'- CCTGGCCAAGGTCATCCATG -3' |
| | reverse 5'- GGAAGGCCATGCCAGTGAGC -3' |
